# Supplementary figures and images for: Reducing publication delay to improve the efficiency and impact of conservation science
Source: PeerJ. 2021 Oct 12;9:e12245. doi: 10.7717/peerj.12245 (PMC8519180; doi:10.7717/peerj.12245)

Synopsis

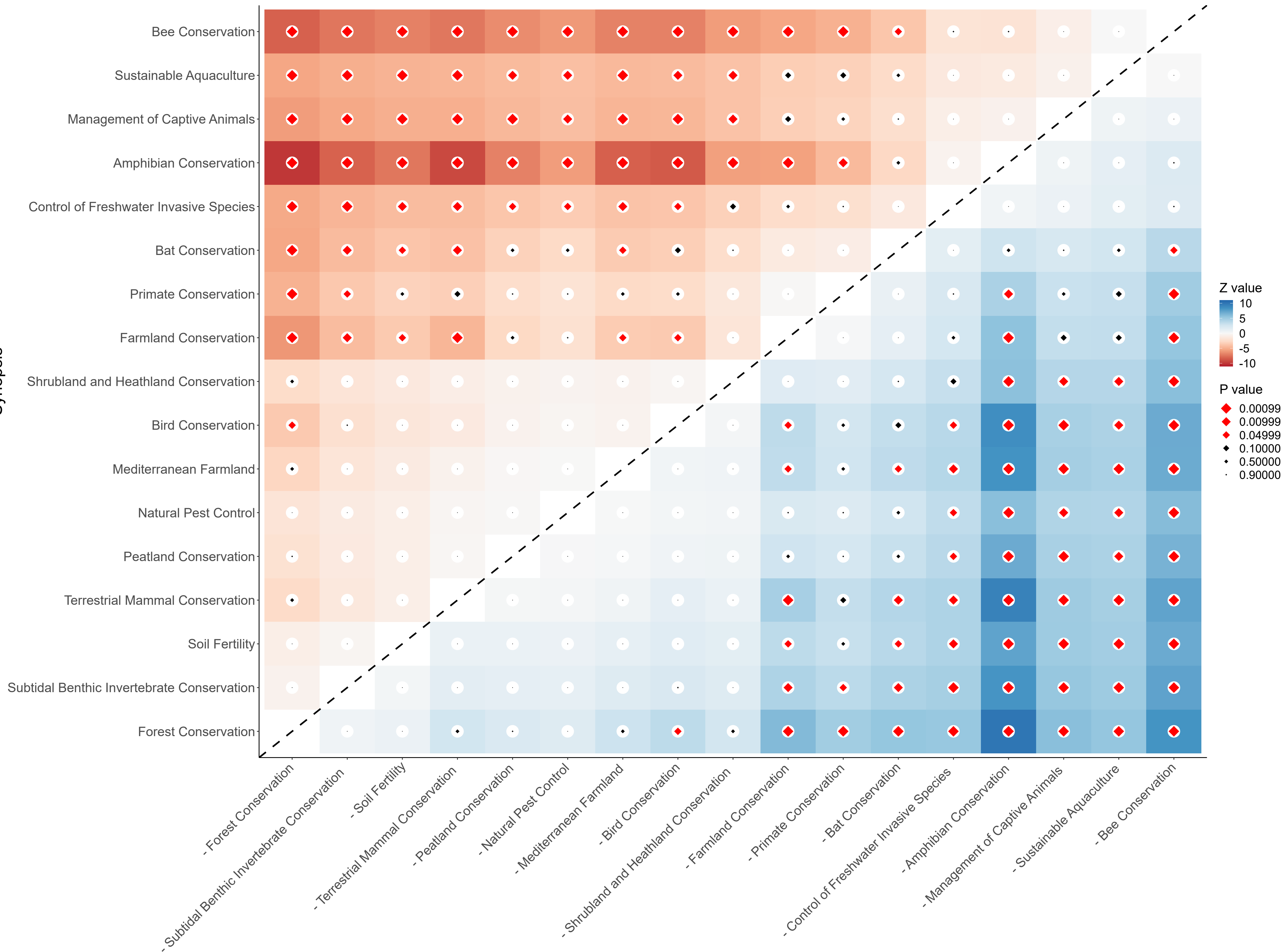

Paired synopsis

Supplement: Supplemental Information 1 — Darker red or blue coloured cells indicate greater Z-ratios (blue = positive difference, red = negative difference) and larger diamonds indicate smaller p-values, whilst red coloured diamonds indicate p-values of p < 0.05 (black diamonds indicate p ≥ 0.05). For example, studies from the synopsis ‘Bee Conservation’ had a significantly shorter mean delay than studies from ‘Forest Conservation’ (top row, first column is dark red with a large red diamond), but did not have a significantly shorter mean delay compared to studies from ‘Sustainable Aquaculture’ (top row, second column from right is grey with a very small black diamond). Post-hoc tests of differences between synopses were conducted using Estimated Marginal Means with Tukey adjustment in the R package emmeans (Lenth, 2021, see main text). [file peerj-09-12245-s001.pdf]

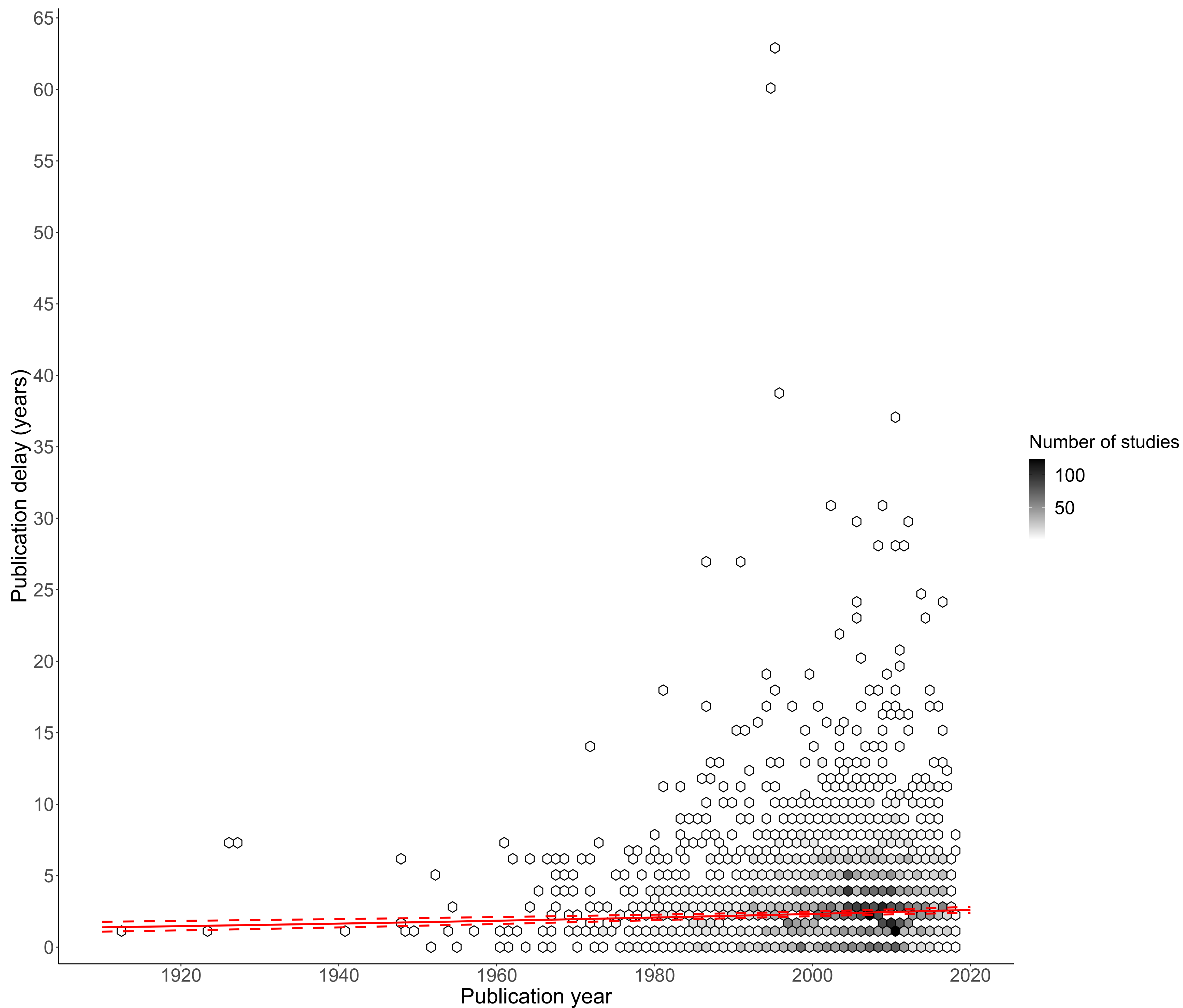

Supplement: Supplemental Information 2 — The shade of hexagons is relative to the number of data points (studies) at that position on the graph. The red solid and dotted lines represent Estimated Marginal Means (averaging over other explanatory variables) and associated 95% confidence intervals based on a quasi–Poisson Generalised Linear Model (GLM) for publication delay (see Table S8 for full model result). This supplemental figure presents all data used in the study (as opposed to Fig. 2, main text, which only visualises studies with a publication delay of 20 years or less). We conducted sensitivity analyses to check whether the trend changed in more recent decades (see Table S4). [file peerj-09-12245-s002.pdf]

# Amphibians, Birds, and Mammals

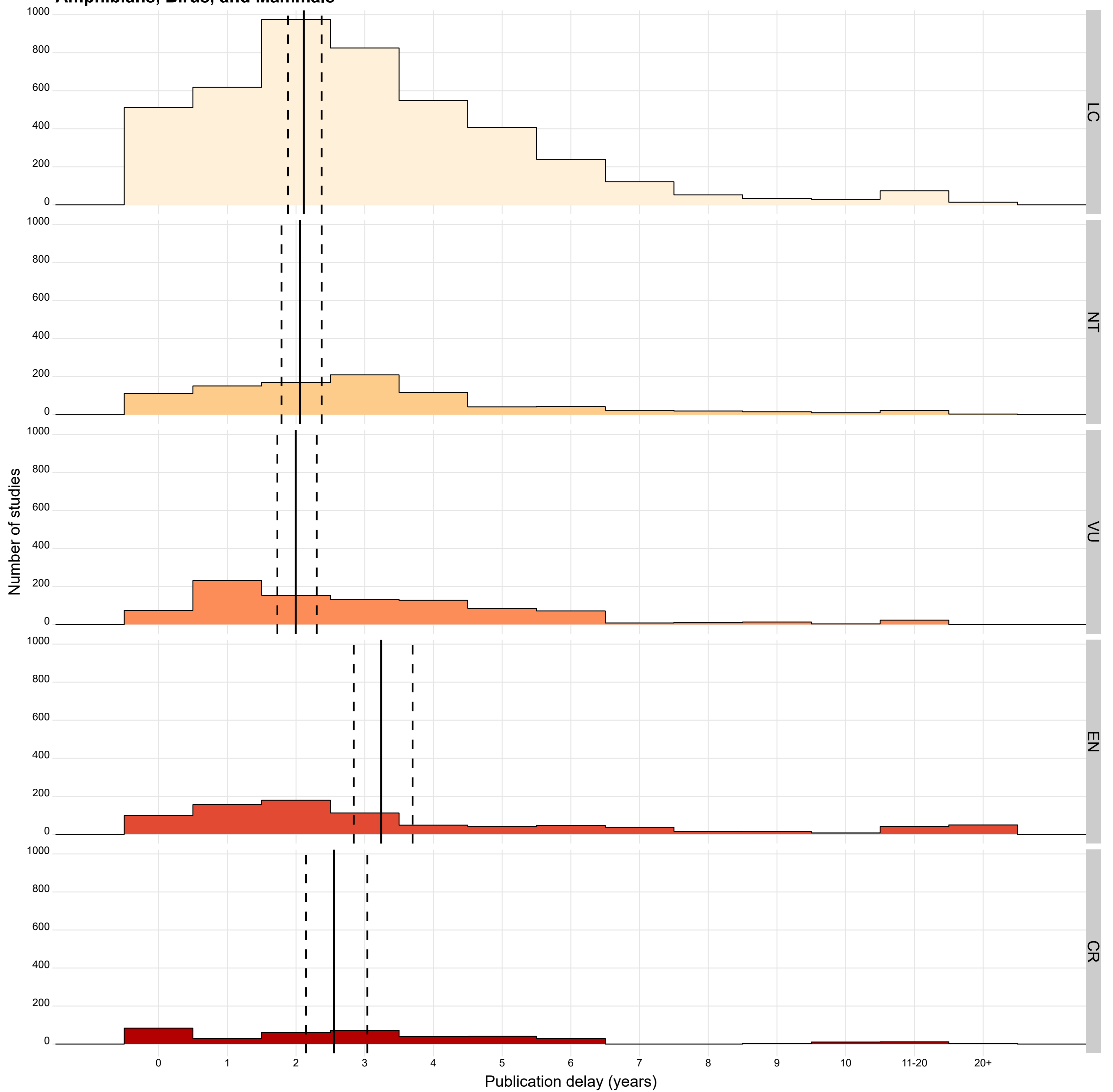

Supplement: Supplemental Information 3 — Data presented for Amphibians (Amphibia from the Amphibian Conservation synopsis), Birds (Aves from the Bird Conservation synopsis), and Mammals (Mammalia from the Bat Conservation, Primate Conservation, and Terrestrial Mammal Conservation synopses) combined. IUCN threatened categories include Vulnerable, Endangered, and Critically Endangered, whilst non-threatened categories include Least Concern and Near Threatened (following IUCN Red List; 2020). We did not include the few studies on Data Deficient and Extinct in the Wild species (see Methods). Vertical solid lines show mean publication delay and dashed lines show 95% Confidence Intervals. Summary estimates were obtained using Estimated Marginal Means (averaging over other explanatory variables) based on quasi–Poisson Generalised Linear Models (GLMs). [file peerj-09-12245-s003.pdf]
