# Supplementary material for: Reducing publication delay to improve the efficiency and impact of conservation science
Source: PeerJ. 2021 Oct 12;9:e12245. doi: 10.7717/peerj.12245 (PMC8519180; doi:10.7717/peerj.12245)
Supplement: Supplemental Information 4 [file peerj-09-12245-s004.docx]

Table S1 — Peer-reviewed and non-peer-reviewed publication sources analysed in the current paper containing studies in the Conservation Evidence database.

| **Peer-reviewed** |
| --- |
| Accident Analysis And Prevention |
| Acta Academiae Agriculturae Ac Technicae Olstenensis, Agricultura |
| Acta Agriculturae Scandinavica — Section B Soil And Plant Science |
| Acta Biologica Slovenica |
| Acta Botanica Neerlandica |
| Acta Chiropterologica |
| Acta Herpetologica |
| Acta Horticulturae |
| Acta Hydrobiologica Sinica |
| Acta Jutlandica |
| Acta Oecologica |
| Acta Phytopathologica Et Entomologica Hungarica |
| Acta Theriologica Sinica |
| African Entomology Memoir |
| African Journal Of Ecology |
| African Journal Of Herpetology |
| African Journal Of Marine Science |
| African Primates |
| Agrarforschung Schweiz |
| Agricultural And Forest Entomology |
| Agricultural Water Management |
| Agriculture, Ecosystems And Environment |
| Agroecology And Sustainable Food Systems |
| Agroforestry Forum |
| Agroforestry Systems |
| Agronomy For Sustainable Development |
| Agronomy Journal |
| Agronomy Research |
| Airo |
| Alces |
| Állattani Közlemények |
| Ambio |
| American Bee Journal |
| American Journal Of Botany |
| American Journal Of Enology And Viticulture |
| American Journal Of Potato Research |
| American Journal Of Primatology |
| American Midland Naturalist |
| Amphibia — Reptilia |
| Amphibian & Reptile Conservation |
| Animal |
| Animal Behaviour |
| Animal Biology |
| Animal Conservation |
| Animal Welfare |
| Annales Botanici Fennici |
| Annales De Limnologie |
| Annales Zoologici Fennici |
| Annals Of Applied Biology |
| Annals Of Botany |
| Annals Of Forest Science |
| Anthrozoos |
| Anzeiger Fur Schadlingskunde |
| Apidologie |
| Applied Animal Behaviour Science |
| Applied Entomology And Zoology |
| Applied Herpetology |
| Applied Soil Ecology |
| Applied Vegetation Science |
| Aquacultural Engineering |
| Aquaculture |
| Aquaculture International |
| Aquaculture Nutrition |
| Aquaculture Research |
| Aquatic Conservation: Marine And Freshwater Ecosystems |
| Aquatic Ecology |
| Aquatic Ecosystem Health & Management |
| Aquatic Invasions |
| Aquatic Living Resources |
| Archives Of Environmental Contamination And Toxicology |
| Arctic, Antarctic, And Alpine Research |
| Ardea |
| Auk |
| Austral Ecology |
| Australian Forestry |
| Australian Journal Of Experimental Agriculture |
| Australian Journal Of Experimental Agriculture And Animal Husbandry |
| Australian Journal Of Zoology |
| Australian Mammalogy |
| Australian Zoologist |
| Barbastella |
| Basic And Applied Ecology |
| Bat Research News |
| Bee World |
| Bellbird |
| Bioagro |
| Biocontrol Science And Technology |
| Biodiversity And Conservation |
| Biodiversity And Insect Pests: Key Issues For Sustainable Management |
| Biofouling |
| Biological Agriculture And Horticulture |
| Biological Bulletin |
| Biological Conservation |
| Biological Control |
| Biological Invasions |
| Biological Journal Of The Linnean Society |
| Biology And Environment |
| Biology And Fertility Of Soils |
| Biology Letters |
| Biomass And Bioenergy |
| Bioresource Technology |
| Bioscience |
| Biota |
| Biota Neotropica |
| Biotropica |
| Bird Conservation International |
| Bird Study |
| BMC Evolutionary Biology |
| Boletin De Sanidad Vegetal, Plagas |
| Bollettino Dell'istituto Di Entomologia Della Università Di Bologna |
| Botanica Helvetica |
| Botanical Journal Of Scotland |
| Botany |
| Braunschweiger Naturkundliche Schriften |
| Brazilian Journal Of Biology |
| British Birds |
| British Herpetological Society Bulletin |
| British Journal Of Entomology And Natural History |
| British Sugar Beet Review |
| British Wildlife |
| Bryologist |
| Bulletin De La Societe Herpetologique De France |
| Bulletin Français De La Píache Et De La Pisciculture |
| Bulletin OEPP |
| Bulletin Of Entomological Research |
| Bulletin Of The Association Of Reptilian And Amphibian Veterinarians |
| Bulletin Of The Maryland Herpetological Society |
| Bulletin Of The Virginia Water Resources Center |
| California Agriculture |
| California Fish And Game |
| Canadian Entomologist |
| Canadian Field-Naturalist |
| Canadian Journal Of Fisheries And Aquatic Sciences |
| Canadian Journal Of Forest Research |
| Canadian Journal Of Plant Pathology |
| Canadian Journal Of Soil Science |
| Canadian Journal Of Zoology |
| Captive Management And Conservation Of Amphibians And Reptiles, Contributions To Herpetology Vol. 11, Society For The Study Of Amphibians And Reptiles |
| Catena |
| Centre For Evidence-Based Conservation |
| Chiroptera Neotropical |
| Cities And The Environment |
| Collaboration For Environmental Evidence |
| Community Ecology |
| Comparative Biochemistry And Physiology. Part A, Molecular & Integrative Physiology |
| Comparative Medicine |
| Condor |
| Conservation Biology |
| Conservation Evidence |
| Conservation Genetics |
| Conservation Letters |
| Conservation Science Western Australia |
| Copeia |
| Coral Reefs |
| Corella |
| Crop Protection |
| Crustaceana |
| Cryobiology |
| Cryo-Letters |
| De Levende Natuur |
| Desert Bighorn Council Transactions |
| Diseases Of Aquatic Organisms |
| Diversity |
| Diversity And Distributions |
| Dodo |
| Ecography |
| Ecohealth |
| Ecología |
| Ecological Applications |
| Ecological Economics |
| Ecological Engineering |
| Ecological Entomology |
| Ecological Indicators |
| Ecological Management & Restoration |
| Ecological Research |
| Ecological Restoration |
| Ecology |
| Ecology And Evolution |
| Ecology And Society |
| Ecology Letters |
| Ecology, Environment And Conservation |
| Ecosphere |
| Ecosystems |
| Emu |
| Endangered Species Bulletin |
| Endangered Species Research |
| Endangered Species Update |
| Entomologia Experimentalis Et Applicata |
| Entomologia Generalis |
| Entomologica Fennica |
| Entomologist's Gazette |
| Entomophaga |
| Environmental Conservation |
| Environmental Entomology |
| Environmental Evidence |
| Environmental Management |
| Environmental Pollution |
| Environmental Science And Pollution Research |
| Environmental Toxicology And Chemistry |
| Environmental Toxicology And Pharmacology |
| EPOPS |
| Estuarine, Coastal And Shelf Science |
| Ethology Ecology And Evolution |
| European Journal Of Agronomy |
| European Journal Of Forest Research |
| European Journal Of Soil Biology |
| European Journal Of Soil Science |
| European Journal Of Wildlife Research |
| Field Crops Research |
| Field Studies |
| Fire Ecology |
| Fisheries Management And Ecology |
| Fisheries Research |
| Florida Scientist |
| Folia Geobotanica |
| Folia Horticulturae |
| Folia Primatologica |
| Folia Zoologica |
| Forest Ecology And Management |
| Forest Science |
| Forest Systems |
| Forestry |
| Forestry Chronicle |
| Freshwater Biology |
| Frontiers In Earth Science |
| Frontiers In Ecology And Evolution |
| Frontiers In Ecology And The Environment |
| Functional Ecology |
| Galemys |
| Genesis |
| Genetics And Molecular Research |
| Geoderma |
| Gibbon Journal |
| Gibier Faune Sauvage, Game Wildlife |
| Global Change Biology |
| Global Ecology And Conservation |
| Gorilla Journal |
| Grass And Forage Science |
| Grassland Science |
| Great Lakes Entomologist |
| HAYATI Journal Of Biosciences |
| Herpetofauna |
| Herpetologica |
| Herpetologica Bonnensis |
| Herpetological Bulletin |
| Herpetological Conservation And Biology |
| Herpetological Journal |
| Herpetological Review |
| Herpetological Society Bulletin |
| Hormones And Behavior |
| Hortscience |
| Horttechnology |
| Human-Wildlife Interactions |
| Hydrobiologia |
| Hystrix |
| Ibis |
| ICES Journal Of Marine Science |
| Insect Conservation And Diversity |
| Insect Science |
| Insectes Sociaux |
| Integrated Pest Management Reviews |
| International Agrophysics |
| International Biodeterioration And Biodegradation |
| International Journal Of Ecology |
| International Journal Of Pest Management |
| International Journal Of Primatology |
| International Journal Of Tropical Insect Science |
| International Journal Of Wildland Fire |
| International Review Of Hydrobiology |
| International Zoo Yearbook |
| Invasive Plant Science And Management |
| IOBC/WPRS Bulletin |
| ISME Journal |
| Japanese Journal Of Applied Entomology And Zoology |
| Journal For Nature Conservation |
| Journal For Nature Conservation And Applied Landscape Ecology |
| Journal Of Agricultural And Food Chemistry |
| Journal Of Agricultural Science |
| Journal Of Agriculture And Social Sciences |
| Journal Of Agronomy And Crop Science |
| Journal Of Animal And Plant Sciences |
| Journal Of Animal Ecology |
| Journal Of Animal Science |
| Journal Of Apicultural Research |
| Journal Of Applied Animal Welfare Science |
| Journal Of Applied Ecology |
| Journal Of Applied Entomology |
| Journal Of Aquatic Plant Management |
| Journal Of Arachnology |
| Journal Of Arid Environments |
| Journal Of Avian Biology |
| Journal Of Bat Research And Conservation |
| Journal Of Chemical Ecology |
| Journal Of Coastal Conservation |
| Journal Of Coastal Research |
| Journal Of Ecology |
| Journal Of Economic Entomology |
| Journal Of Entomological Science |
| Journal Of Environmental Education |
| Journal Of Environmental Management |
| Journal Of Environmental Planning And Management |
| Journal Of Environmental Quality |
| Journal Of Environmental Studies |
| Journal Of Ethology |
| Journal Of Experimental Botany |
| Journal Of Experimental Zoology Part A: Ecological And Integrative Physiology |
| Journal Of Field Ornithology |
| Journal Of Fish And Wildlife Management |
| Journal Of Fish Biology |
| Journal Of Fish Diseases |
| Journal Of Food, Agriculture And Environment |
| Journal Of Forest Research |
| Journal Of Forest Science |
| Journal Of Forestry Research |
| Journal Of Herpetological Medicine And Surgery |
| Journal Of Herpetology |
| Journal Of Hymenoptera Research |
| Journal Of Insect Conservation |
| Journal Of Insect Science |
| Journal Of Invertebrate Pathology |
| Journal Of Mammalogy |
| Journal Of Molluscan Studies |
| Journal Of Natural History |
| Journal Of Nematology |
| Journal Of Ornithology |
| Journal Of Pest Science |
| Journal Of Pharmacological And Toxicological Methods |
| Journal Of Plant Diseases And Protection |
| Journal Of Raptor Research |
| Journal Of Sea Research |
| Journal Of Soils And Sediments |
| Journal Of Soils And Water Conservation |
| Journal Of Sustainable Forestry |
| Journal Of The Acoustical Society Of America |
| Journal Of The American Society For Horticultural Science |
| Journal Of The Elisha Mitchell Scientific Society |
| Journal Of The Iowa Academy Of Science |
| Journal Of The Kansas Entomological Society |
| Journal Of The Marine Biological Association Of The United Kingdom |
| Journal Of The Torrey Botanical Society |
| Journal Of Threatened Taxa |
| Journal Of Tropical Ecology |
| Journal Of Vegetation Science |
| Journal Of Wetlands Environmental Management |
| Journal Of Wildlife Diseases |
| Journal Of Wildlife Management |
| Journal Of Wildlife Rehabilitation |
| Journal Of Zoo And Aquarium Research |
| Journal Of Zoo And Aquatic Research |
| Journal Of Zoo And Wildlife Medicine |
| Journal Of Zoology |
| Kasetsart Journal — Natural Science |
| Koedoe |
| Laboratory Animals |
| Lake And Reservoir Management |
| Land Degradation & Development |
| Landscape And Urban Planning |
| Landscape Ecology |
| Lemur News |
| Lichenologist |
| Limnology |
| Limosa |
| Lutra |
| Malayan Nature Journal |
| Mammal Research |
| Mammal Review |
| Mammal Study |
| Mammalia |
| Mammalian Biology |
| Management Of Biological Invasions |
| Marine And Freshwater Research |
| Marine Ecology |
| Marine Ecology — Progress Series |
| Marine Environmental Research |
| Marine Mammal Science |
| Marine Ornithology |
| Marine Policy |
| Marine Pollution Bulletin |
| Mastozoologia Neotropical |
| Medical And Veterinary Entomology |
| Medical Mycology |
| Memoirs Of The Queensland Museum |
| Memoranda — Societatis Pro Fauna Et Flora Fennica |
| Microbial Ecology |
| Mires And Peat |
| Mitteilungen Der Biologischen Bundesanstalt Für Land-U. Forstwirtschaft |
| Mitteilungen Der Deutschen Gesellschaft Fuer Allgemeine Und Angewandte Entomologie |
| Molecular Ecology |
| Munibe Antropologia-Arkeologia |
| Mycorrhiza |
| Natur Und Landschaft |
| Natura Societa Italiana Di Scienze Naturale E Museo Civico Di Storia Naturale Milan |
| Natural Areas Journal |
| Nature |
| Neotropical Primates |
| Neurobiology Of Learning And Memory |
| New Forests |
| New Zealand Journal Of Botany |
| New Zealand Journal Of Crop And Horticultural Science |
| New Zealand Journal Of Zoology |
| New Zealand Plant Protection |
| North American Journal Of Aquaculture |
| North American Journal Of Fisheries Management |
| Northeastern Naturalist |
| Northwest Science |
| Notulae Botanicae Horti Agrobotanici Cluj-Napoca |
| Nutrient Cycling In Agroecosystems |
| Oceanographic Literature Review |
| Oecologia |
| Ohio Journal Of Sciences |
| Oikos |
| Oncoscience |
| Organic Agriculture |
| Ornis Svecica |
| Ornithologische Beobachter |
| Ornitologia Colombiana |
| Ornitologia Neotropical |
| Oryx |
| Outlook On Agriculture |
| Pachyderm |
| Pacific Conservation Biology |
| Pacific Science |
| Paddy And Water Environment |
| Pakistan Journal Of Agricultural Sciences |
| Pakistan Journal Of Biological Sciences |
| Pakistan Journal Of Scientific And Industrial Research Series B: Biological Sciences |
| Peanut Science |
| Pedobiologia |
| Perspectives In Plant Ecology, Evolution And Systematics |
| Pest Management Science |
| Physiological Entomology |
| Phyton (Horn) |
| Phytoparasitica |
| Phytoprotection |
| Pianura |
| Plant And Soil |
| Plant Breeding |
| Plant Disease |
| Plant Ecology |
| Plant Ecology And Diversity |
| Plant Ecology And Evolution |
| Plant Health Progress |
| Plant Systematics And Evolution |
| Planta Daninha |
| Plos ONE |
| Polar Biology |
| Polish Journal Of Ecology |
| Population Ecology |
| Preslia |
| Primate Conservation |
| Primates |
| Procedia Environmental Sciences |
| Proceedings Of The Indian Academy Of Sciences |
| Proceedings Of The Latvian Academy Of Sciences, Section B: Natural, Exact, And Applied Sciences |
| Proceedings Of The National Academy Of Sciences Of The United States Of America |
| Proceedings Of The Royal Society B: Biological Sciences |
| Progress In Materials Science |
| Rangeland Ecology & Management |
| RAVON |
| Renewable Agriculture And Food Systems |
| Reproduction |
| Reproduction, Fertility And Development |
| Reproductive Biology And Endocrinology |
| Research In Veterinary Science |
| Restoration Ecology |
| Revista Brasileira De Ciencia Do Solo |
| Revista Brasileira De Entomologia |
| Revista De Biologia Tropical |
| Revue d'Ecologie (La Terre Et La Vie) |
| River Research And Applications |
| Royal Society Open Science |
| Russian Journal Of Ecology |
| Salamandra |
| Scandinavian Journal Of Forest Research |
| Schweiz. Peckiana |
| Science |
| Science For Conservation |
| Scienceasia |
| Scientia Marina |
| Scientific Proceedings Of The Royal Dublin Society, Series A |
| Scientific Reports |
| Seabird Bycatch: Trends, Roadblocks And Solutions |
| Slovak Raptor Journal |
| Sociobiology |
| Soil And Tillage Research |
| Soil Biology And Biochemistry |
| Soil Research |
| Soil Science Society Of America Journal |
| Soil Use And Management |
| South African Journal Of Botany |
| South African Journal Of Enology And Viticulture |
| South African Journal Of Plant And Soil |
| South African Journal Of Wildlife Research |
| Southeastern Naturalist |
| Southwestern Naturalist |
| Strix |
| Studies On Neotropical Fauna And Environment |
| Sustainability Science |
| Swedish Journal Of Agricultural Research |
| Systematic Review No. 11. Collaboration For Environmental Evidence / Centre For Evidence-Based Conservation |
| Tearmann |
| Technical Bulletin, Agricultural Experiment Station, University Of Maine |
| The Scientific World Journal |
| Theriogenology |
| Therya |
| Transactions Of The Illinois State Academy Of Science |
| Transactions Of The Missouri Academy Of Science |
| Transactions Of The Western Section Of The Wildlife Society |
| Transportation Research Record |
| Tropical Conservation Science |
| Tropical Ecology |
| Tuexenia |
| Turk Tarim Ve Ormancilik Dergisi/Turkish Journal Of Agriculture And Forestry |
| Urban Ecosystems |
| Ursus |
| Vaccine |
| Vagos |
| Victorian Naturalist |
| Virginia Journal Of Science |
| WAIT School Of Biology Bulletin |
| Water Science And Technology |
| Waterbirds |
| Weed Biology And Management |
| Weed Research |
| Weed Science |
| Weed Technology |
| Western North American Naturalist |
| Wetlands |
| Wetlands Ecology And Management |
| Wildlife Biology |
| Wildlife Research |
| Wildlife Society Bulletin |
| Wilson Journal Of Ornithology |
| Zeitschrift Für Angewandte Entomologie |
| Zeitschrift Für Jagdwissenschaft |
| Zeledonia |
| Zemdirbyste |
| Zoo Biology |
| Zoological Science |
| Zoologische Garten |
| Zoology And Ecology |
| **Non-peer-reviewed** |
| 10th Annual Conference Of The International Association For Landscape Ecology |
| 10th International Congress Of Plant Protection: Plant Protection For Human Welfare, 20-25 November, 1983 |
| 130 Proceedings-Vertebrate Pest Conference |
| 15th Meeting Of The European Grassland Federation |
| 1997 Brighton Crop Protection Conference — Weeds, Conference Proceedings |
| 19th Meeting Of The European Grassland Federation |
| 2005 Annual International Research Conference On Methyl Bromide Alternatives And Emissions Reductions, 31st October-3rd November, 2005 |
| 24th Vertebrate Pest Conference |
| 7th International Safflower Conference |
| A Report Submitted To The Bats And Wind Energy Cooperative. |
| Abstracts Of The EWRS-Symposium 2007, Hamar, Norway |
| African Bird Club Bulletin |
| Agrarokologie |
| Alternative Functions Of Grassland. Proceedings Of The 15th European Grassland Federation Symposium, 7-9 September 2009 |
| American Fisheries Society Symposium |
| American Zoo And Aquarium Association Annual Conference Proceedings |
| Amphibian Ark Newsletter |
| Amphibian Declines: The Conservation Status Of United States Species |
| Amphibians And Roads: Proceedings Of The Toad Tunnel Conference |
| Amphibians And Roads: Toad Tunnel Conference |
| Análisis Espacial Y Representación Geográfica: Innovación Et Aplicación |
| Arthropod Natural Enemies In Arable Land |
| Arthropod Natural Enemies In Arable Land I — Density, Spatial Heterogeneity And Dispersal, Acta Jutlandica |
| Avian Landscape Ecology: Pure And Applied Issues In The Large-Scale Ecology Of Birds |
| Bats And Forests Symposium |
| Bats In Captivity Volume 2: Aspects Of Rehabilitation |
| Biodiversity News |
| British Crop Protection Conference: Pests And Diseases |
| British Crop Protection Council Monographs |
| British Grassland Society Fifth Research Conference |
| British Grassland Society Occasional Symposium |
| BTO Research Report |
| Bulletin OILB SROP |
| California Energy Commission Report |
| Carabid Beetles: Ecology And Evolution |
| CEFAS Final Contract Report C5256 |
| Centre For Ecology And Hydrology Defra Project Code PH0422 |
| Changes In The Fauna Of Wild Bees In Europe |
| Coastal Meadow Management — Best Practice Guidelines |
| Conservation And Management Of Great Crested Newts |
| Creating New Habitats In Intensively Used Farmland |
| Declines And Disappearances Of Australian Frogs |
| Defra |
| Department Of Biological Sciences |
| Department Of Ecology & Evolutionary Biology |
| Deutsche Gesellschaft Für Herpetologie Und Terrarienkunde |
| Durrell Institute Of Conservation And Ecology |
| Ecological Society Of America Annual Meeting Abstracts |
| Ecology And Conservation Of Lowland Farmland Birds. Spring Conference Of The British Ornithologists' Union, 27-28 March 1999 |
| Ecology And Integrated Farming Systems |
| Ecosystems And Sustainable Development III, Advances In Ecological Sciences |
| Effect Of Sward Type And Management On Diversity Of Upland Birds. |
| Eighth International Herpetological Symposium |
| Enact |
| Environmental Encounters Series: Workshop On Ecological Corridors For Invertebrates: Strategies Of Dispersal And Recolonisation In Today's Agricultural And Forestry Landscapes |
| European And Mediterranean Plant Protection Organization |
| European And Mediterranean Plant Protection Organization Report Number 09-15078 Rev |
| Exmoor Mires Partnership |
| Fencing For Conservation. Restriction Of Evolutionary Potential Or A Riposte To Threatening Processes? |
| Fibl Dossier |
| Field Margins: Integrating Agriculture And Conservation |
| Forage For Bees In An Agricultural Landscape |
| Forestry Commission Report |
| Forward With Grass Into Europe: British Grassland Society Winter Meeting |
| French Peatland Coordination Centre |
| Froglife Conservation Report No.1 |
| Froglog |
| Frogs In The Community |
| Global Re-Introduction Perspectives: 2008. Re-Introduction Case-Studies From Around The Globe |
| Global Re-Introduction Perspectives: 2010. Additional Case Studies From Around The Globe |
| Global Re-Introduction Perspectives: 2011. More Case Studies From Around The Globe |
| Grassland Science In Europe |
| Grazing Management: The Principles And Practice Of Grazing, For Profit And Environmental Gain, Within Temperate Grassland Systems: Proceedings Of The British Grassland Society Conference, 29 February-2 March, 2000 |
| Habitat Fragmentation & Infrastructure |
| Hedgerows Of The World: Their Ecological Functions In Different Landscapes, International Association For Landscape Ecology, 10th Annual Conference Of The International Association For Landscape Ecology |
| Herpetofauna And Roads Workshop — Is There Light At The End Of The Tunnel? |
| High Value Grassland, British Grassland Society Occasional Symposium No.38 |
| High Value Grassland: Providing Biodiversity, A Clean Environment And Premium Products. British Grassland Society Occasional Symposium No.38 |
| High Value Grassland: Providing Biodiversity, A Clean Environment And Premium Products. . British Grassland Society Occasional Symposium No.38 |
| High Value Grassland: Providing Biodiversity, A Clean Environment And Premium Products, British Grassland Society Occasional Symposium |
| How To Protect Or What We Know About Carabid Beetles: From Knowledge To Application, From Wijster (1969) To Tuczno (2001), 2002 Conference |
| ICROFS News |
| IFOAM 2000: The World Grows Organic |
| In Practice: Bulletin Of The Chartered Institute Of Ecology And Environmental Management |
| Infos-Ctifl |
| Insect Pest Management |
| International Conference On Habitat Fragmentation Due To Transportation Infrastructure |
| International Occasional Symposium Of The European Grassland Federation. |
| International Symposium And Workshop On Tropical Peatland |
| ITE Symposium |
| Joint Meeting Between The British Grassland Society And The British Ecological Society: Grassland Management And Nature Conservation. British Grassland Society Occasional Symposium |
| Kalimantan Forests And Climate Partnership |
| Land Retirement Demonstration Project Five Year Report |
| Landscape Management For Functional Biodiversity 2nd Working Group Meeting. 16-19 May 2006. |
| Landscape Management For Functional Biodiversity, 2nd Working Group Meeting |
| LIFE Project: Anglesey & Llyn Fens |
| Livestock Farming Systems: Integrating Animal Science Advances In The Search Of Sustainability |
| London Naturalist |
| Mededelingen Van De Faculteit Landbouwwetenschappen Universiteit Gent |
| Möglichkeiten Und Grenzen Der Ökologisierung Der Landwirtschaft: Wissenschaftliche Grundlagen Und Praktische Erfahrungen; Beiträge Aus Dem Arbeitskreis |
| National Park Service Point Reyes National Seashore, California, USA |
| Natuurhistorisch Maandblad |
| NERI, Technical Report |
| New Forest Plants Project, UK |
| Our Living Resources: A Report To The Nation On The Distribution, Abundance, And Health Of US Plants, Animals, And Ecosystems |
| Penny Anderson Associates Report |
| Pesticides, Cereal Farming And The Environment: The Boxworth Project |
| Phd Thesis |
| Plant Invasions: General Aspects And Special Problems |
| Plant Research International, Wageningen |
| Primate Tourism: A Tool For Conservation |
| Proceedings — Brighton Crop Protection Conference |
| Proceedings 20th German Conference On Weed Biology And Weed Control |
| Proceedings Of A Symposium On Cheetahs As Game Ranch Animals |
| Proceedings Of National Avian-Wind Power Planning Meeting IV |
| Proceedings Of The 1987 International Crane Workshop International Crane Foundation, |
| Proceedings Of The 1989 American Association Of Zoological Parks And Aquariums National Conference |
| Proceedings Of The 1998 International Conference On Wildlife Ecology And Transportation |
| Proceedings Of The 1999 International Conference On Wildlife Ecology And Transportation |
| Proceedings Of The 2001 International Conference On Ecology And Transportation |
| Proceedings Of The 2003 International Conference On Ecology And Transportation |
| Proceedings Of The 2005 International Conference On Ecology And Transportation |
| Proceedings Of The 2007 International Conference On Ecology And Transportation |
| Proceedings Of The 5th Annual Reptile Symposium On Captive Propagation And Husbandry |
| Proceedings Of The Asian Wetland Symposium |
| Proceedings Of The British Grassland Society/British Ecological Society Conference |
| Proceedings Of The Conservation And Management Of Great Crested Newts |
| Proceedings Of The Eastern Wildlife Damage Control Conference |
| Proceedings Of The Eastern Wildlife Damage Management Conference |
| Proceedings Of The Eleventh Vertebrate Pest Conference |
| Proceedings Of The Fourth International Workshop, 26-29 November 2001 |
| Proceedings Of The Hedgerow Management And Nature Conservation: British Ecological Society Conservation Ecology Group |
| Proceedings Of The HGCA Conference, Arable Crop Protection In The Balance: Profit And The Environment |
| Proceedings Of The International Conference On Wildlife Ecology And Transportation |
| Proceedings Of The International Symposium On Tropical Peatlands |
| Proceedings Of The International Union Of Game Biologists 17th Congress |
| Proceedings Of The Lowland Farmland Birds III: Delivering Solutions In An Uncertain World |
| Proceedings Of The Ninth Wildlife Damage Management Conference |
| Proceedings Of The Relationship Between Nature Conservation, Biodiversity And Organic Agriculture |
| Proceedings Of The Sixteenth Vertebrate Pest Conference |
| Proceedings Of The Sixth Workshop For Tropical Agricultural Entomologists, May 1998 |
| Proceedings Of The South Dakota Academy Of Science |
| Proceedings Of The Tenth Vertebrate Pest Conference |
| Proceedings Of The Thirteenth Australian Weeds Conference |
| Proceedings Of The Trends In Addressing Transportation Related Wildlife Mortality: Transportation Related Wildlife Mortality Seminar, FL-ER-58-96 |
| Proceedings Of The Trends In Addressing Wildlife Mortality: Transportation Related Wildlife Mortality Seminar, FL-ER-58-96 |
| Proceedings The Joint Meeting Between The British Grassland Society And The British Ecological Society. Grassland Management And Nature Conservation |
| Proceedings, Soil And Crop Science Society Of Florida |
| Protecting Threatened Bats At Coal Mines: A Technical Interactive Forum |
| Raised Bog Management For Biological Diversity Conservation In Latvia |
| Recent Developments In Cereal Production. |
| Re-Creating Plant And Beetle Assemblages Of Species-Rich Chalk Grasslands On Ex-Arable Land |
| Regional Meetings Of The American Association Of Zoological Parks And Aquariums |
| Rehabilitation Of Oiled African Penguins: A Conservation Success Story |
| Re-Introduction News |
| RELU Policy And Practice Note, Number 37 Report |
| Rencontre Recherche Ruminants |
| Report For Confederación Hidrográfica Del Ebro |
| Report To Infra Econetwork Europe (IENE), Fifth IENE Meeting |
| Report To INRENA |
| Report To Syngenta |
| Report To The Nature Conservancy Council (GB) |
| Restoration Of Endangered Species: Conceptual Issues, Planning And Implementation |
| Restoration Of Temperate Wetlands |
| RSPB |
| School Of Graduate Studies And Research |
| Scottish Natural Heritage Reports |
| Second International Symposium On Biological Control Of Arthropods, September 12-16, 2005 |
| Simposio: Estado Del Conocimiento De La Biología Reproductiva De Las Especies De Loros Amenazados De Colombia Con Énfasis En Iniciativas De Conservación |
| Simposio: Estado Del Conocimiento De La Biología Reproductiva De Las Especies De Loros Amenazados De Colombia Con Énfasis En Iniciativas De Conservación. II Congreso Colombiano De Zoología |
| Sonoma State University And Marin/Sonoma Mosquito And Vector Control District, Sonoma, California |
| Status And Conservation Of Midwestern Amphibians |
| Suffolk Natural History |
| Terra Australis |
| The Brighton Crop Protection Conference — Weeds |
| The Ecology And Conservation Of Lowland Farmland Birds |
| The Ecology Of Temperate Cereal Fields |
| The humble bee: its life history and how to domesticate it. |
| The National Trust Conservation Newsletter |
| The Structure And Functioning Of Flower-Visiting Insect Communities On Hay Meadows |
| Timber, Tourists And Temples. Conservation And Development In The Maya Forest Of Belize, Guatemala And Mexico |
| Tropical Peat Swamp Forest Silviculture In Central Kalimantan |
| TWSG News |
| Unpublished Report Commissioned By The National Trust |
| Unpublished Report To The West Sussex Heathlands Project |
| Urban Herpetology |
| USDA-ARS-SABCL (South American Biological Control Laboratory). Annual Report, 2011 |
| Use Of The Tubingen Mix For Bee Pasture In Germany |
| Verhandlungen Der Gesellschaft Fur Okologie |
| Vingtième Conférence Du Columa Journées Internationales Sur La Lutte Contre Les Mauvaises Herbes |
| Wading Birds |
| Washington Sea Grant Program, University Of Washington Report |
| Western Foundation Of Vertebrate Zoology |
| Wolves: Behavior, Ecology, And Conservation |
| ZEF Bonn |
