# Supplementary material for: Reducing publication delay to improve the efficiency and impact of conservation science
Source: PeerJ. 2021 Oct 12;9:e12245. doi: 10.7717/peerj.12245 (PMC8519180; doi:10.7717/peerj.12245)
Supplement: Supplemental Information 7 — The synopsis ‘Management of Captive Animals’ and publication source ‘non-journal literature’ were set to the zero value of the beta slope and used as reference categories. Values of 0.000 represent values less than 0.001. [file peerj-09-12245-s007.docx]

Table S4 — Results of sensitivity analysis using quasi-Poisson Generalised Linear Models (GLM) for different time periods to assess whether the fact that more recently published studies are more likely to have a longer delay may have affected our results (see Methods). The synopsis ‘Management of Captive Animals’ and publication source ‘non-journal literature’ were set to the zero value of the beta slope and used as reference categories. Values of 0.000 represent values less than 0.001.

| 1980-2020 | | | | |
| --- | --- | --- | --- | --- |
| Parameter | Estimate | Standard Error | t-value | p-value |
| Intercept | -10.81 | 3.035 | -3.562 | 0 |
| Publication Delay | 0.005 | 0.002 | 3.593 | 0 |
| Bird Conservation | 0.712 | 0.103 | 6.879 | 0 |
| Farmland Conservation | 0.564 | 0.106 | 5.344 | 0 |
| Natural Pest Control | 0.733 | 0.123 | 5.968 | 0 |
| Control of Freshwater Invasive Species | 0.306 | 0.145 | 2.106 | 0.035 |
| Shrubland and Heathland Conservation | 0.688 | 0.119 | 5.802 | 0 |
| Terrestrial Mammal Conservation | 0.768 | 0.104 | 7.394 | 0 |
| Bat Conservation | 0.478 | 0.121 | 3.965 | 0 |
| Amphibian Conservation | 0.241 | 0.112 | 2.143 | 0.032 |
| Forest Conservation | 0.889 | 0.109 | 8.18 | 0 |
| Primate Conservation | 0.562 | 0.114 | 4.934 | 0 |
| Peatland Conservation | 0.741 | 0.114 | 6.477 | 0 |
| Mediterranean Farmland | 0.735 | 0.106 | 6.924 | 0 |
| Subtidal Benthic Invertebrate Conservation | 0.848 | 0.115 | 7.381 | 0 |
| Management of Captive Animals | 0.157 | 0.156 | 1.007 | 0.314 |
| Soil Fertility | 0.829 | 0.118 | 7.031 | 0 |
| Sustainable Aquaculture | 0.03 | 0.184 | 0.16 | 0.873 |
| Peer-review (yes) | 0.438 | 0.065 | 6.715 | 0 |
| 1990-2020 | | | | |
| Intercept | -9.866 | 3.82 | -2.583 | 0.01 |
| Publication Delay | 0.005 | 0.002 | 2.606 | 0.009 |
| Bird Conservation | 0.687 | 0.104 | 6.594 | 0 |
| Farmland Conservation | 0.541 | 0.106 | 5.09 | 0 |
| Natural Pest Control | 0.713 | 0.126 | 5.683 | 0 |
| Control of Freshwater Invasive Species | 0.306 | 0.146 | 2.089 | 0.037 |
| Shrubland and Heathland Conservation | 0.685 | 0.12 | 5.725 | 0 |
| Terrestrial Mammal Conservation | 0.759 | 0.105 | 7.241 | 0 |
| Bat Conservation | 0.471 | 0.122 | 3.88 | 0 |
| Amphibian Conservation | 0.234 | 0.113 | 2.068 | 0.039 |
| Forest Conservation | 0.878 | 0.109 | 8.035 | 0 |
| Primate Conservation | 0.588 | 0.115 | 5.12 | 0 |
| Peatland Conservation | 0.727 | 0.115 | 6.312 | 0 |
| Mediterranean Farmland | 0.724 | 0.107 | 6.785 | 0 |
| Subtidal Benthic Invertebrate Conservation | 0.841 | 0.115 | 7.282 | 0 |
| Management of Captive Animals | 0.142 | 0.157 | 0.904 | 0.366 |
| Soil Fertility | 0.827 | 0.118 | 6.988 | 0 |
| Sustainable Aquaculture | 0.017 | 0.184 | 0.092 | 0.927 |
| Peer-review (yes) | 0.461 | 0.068 | 6.755 | 0 |
| 2000-2020 | | | | |
| Intercept | -3.441 | 5.762 | -0.597 | 0.55 |
| Publication Delay | 0.002 | 0.003 | 0.589 | 0.556 |
| Bird Conservation | 0.826 | 0.12 | 6.881 | 0 |
| Farmland Conservation | 0.713 | 0.122 | 5.854 | 0 |
| Natural Pest Control | 0.91 | 0.143 | 6.347 | 0 |
| Control of Freshwater Invasive Species | 0.316 | 0.166 | 1.904 | 0.057 |
| Shrubland and Heathland Conservation | 0.877 | 0.134 | 6.534 | 0 |
| Terrestrial Mammal Conservation | 0.931 | 0.12 | 7.747 | 0 |
| Bat Conservation | 0.641 | 0.134 | 4.794 | 0 |
| Amphibian Conservation | 0.431 | 0.128 | 3.366 | 0.001 |
| Forest Conservation | 1.067 | 0.123 | 8.64 | 0 |
| Primate Conservation | 0.849 | 0.129 | 6.587 | 0 |
| Peatland Conservation | 0.906 | 0.13 | 6.985 | 0 |
| Mediterranean Farmland | 0.905 | 0.121 | 7.453 | 0 |
| Subtidal Benthic Invertebrate Conservation | 1.015 | 0.128 | 7.901 | 0 |
| Management of Captive Animals | 0.282 | 0.184 | 1.529 | 0.126 |
| Soil Fertility | 1 | 0.133 | 7.542 | 0 |
| Sustainable Aquaculture | 0.172 | 0.191 | 0.899 | 0.369 |
| Peer-review (yes) | 0.449 | 0.078 | 5.724 | 0 |
| 2010-2020 | | | | |
| Intercept | -13.273 | 20.151 | -0.659 | 0.51 |
| Publication Delay | 0.007 | 0.01 | 0.728 | 0.467 |
| Bird Conservation | -0.38 | 0.513 | -0.742 | 0.458 |
| Farmland Conservation | -0.508 | 0.514 | -0.99 | 0.322 |
| Natural Pest Control | -0.263 | 0.532 | -0.494 | 0.621 |
| Control of Freshwater Invasive Species | -1.026 | 0.531 | -1.931 | 0.054 |
| Shrubland and Heathland Conservation | -0.355 | 0.514 | -0.69 | 0.49 |
| Terrestrial Mammal Conservation | -0.246 | 0.507 | -0.486 | 0.627 |
| Bat Conservation | -0.557 | 0.513 | -1.086 | 0.277 |
| Amphibian Conservation | -0.941 | 0.515 | -1.828 | 0.068 |
| Forest Conservation | 0.069 | 0.509 | 0.135 | 0.893 |
| Primate Conservation | -0.333 | 0.511 | -0.652 | 0.514 |
| Peatland Conservation | -0.301 | 0.511 | -0.59 | 0.556 |
| Mediterranean Farmland | -0.386 | 0.507 | -0.761 | 0.447 |
| Subtidal Benthic Invertebrate Conservation | -0.209 | 0.511 | -0.409 | 0.682 |
| Management of Captive Animals | -0.896 | 0.535 | -1.675 | 0.094 |
| Soil Fertility | -0.307 | 0.517 | -0.595 | 0.552 |
| Sustainable Aquaculture | -1.14 | 0.569 | -2.005 | 0.045 |
| Peer-review (yes) | 0.216 | 0.126 | 1.716 | 0.086 |
