# Supplementary material for: Reducing publication delay to improve the efficiency and impact of conservation science
Source: PeerJ. 2021 Oct 12;9:e12245. doi: 10.7717/peerj.12245 (PMC8519180; doi:10.7717/peerj.12245)
Supplement: Supplemental Information 10 — Selected models are identical to those selected for the main taxonomic analyses (see Table S5). Publication delay (difference between year data collection ended and year study was published) was the response variable for all models. [file peerj-09-12245-s010.docx]

Table S7 — Selection of model structure by qAIC ranking for taxonomic sensitivity analyses (selecting a random species instead of the most threatened species from each study). Selected models are identical to those selected for the main taxonomic analyses (see Table S5). Publication delay (difference between year data collection ended and year study was published) was the response variable for all models.

| **All taxa (amphibians, birds, and mammals)** | | | | | |
| --- | --- | --- | --- | --- | --- |
| Model rank | Model structure | df | logLik | qAIC | delta |
| Selected | Delay ~ peer-review + publication date + synopsis + IUCN Red List category | 11 | -20068 | 11038 | 0 |
| A | Delay ~ peer-review + synopsis + IUCN Red List category | 10 | -20105 | 11057 | 18.3 |
| B | Delay ~ publication date + synopsis + IUCN Red List category | 10 | -20153 | 11083 | 44.8 |
| C | Delay ~ synopsis + IUCN Red List category | 9 | -20195 | 11105 | 66.1 |
| D | Delay ~ peer-review + publication date + synopsis | 7 | -20345 | 11183 | 144.5 |
| E | Delay ~ peer-review + synopsis | 6 | -20386 | 11203 | 164.6 |
| F | Delay ~ publication date + synopsis | 6 | -20437 | 11231 | 192.9 |
| G | Delay ~ synopsis | 5 | -20484 | 11255 | 216.5 |
| H | Delay ~ peer-review + publication date + IUCN Red List category | 7 | -20497 | 11266 | 227.8 |
| I | Delay ~ peer-review + IUCN Red List category | 6 | -20531 | 11283 | 244.3 |
| J | Delay ~ publication date + IUCN Red List category | 6 | -20630 | 11337 | 298.9 |
| K | Delay ~ IUCN Red List category | 5 | -20668 | 11356 | 317.5 |
| L | Delay ~ peer-review + publication date | 3 | -20798 | 11423 | 384.6 |
| M | Delay ~ peer-review | 2 | -20841 | 11445 | 406.5 |
| N | Delay ~ publication date | 2 | -20929 | 11493 | 454.8 |
| O | Delay ~ 1 | 1 | -20977 | 11517 | 478.9 |
| **Amphibians** | | | | | |
| Model rank | Model structure | df | logLik | qAIC | delta |
| Selected | Delay ~ peer-review + publication date | 3 | -1586 | 1340 | 0 |
| A | Delay ~ publication date | 2 | -1595 | 1345 | 5.1 |
| B | Delay ~ peer-review | 2 | -1595 | 1346 | 5.8 |
| C | Delay ~ peer-review + publication date + IUCN Red List category | 7 | -1585 | 1347 | 6.8 |
| D | Delay ~ 1 | 1 | -1604 | 1351 | 11.1 |
| E | Delay ~ peer-review + IUCN Red List category | 6 | -1593 | 1351 | 11.4 |
| F | Delay ~ publication date + IUCN Red List category | 6 | -1593 | 1352 | 12.1 |
| G | Delay ~ IUCN Red List category | 5 | -1602 | 1357 | 17.2 |
| **Birds** | | | | | |
| Model rank | Model structure | df | logLik | qAIC | delta |
| Selected | Delay ~ IUCN Red List category | 5 | -8424 | 5162 | 0 |
| A | Delay ~ publication date + IUCN Red List category | 6 | -8423 | 5163 | 1.3 |
| B | Delay ~ peer-review + IUCN Red List category | 6 | -8423 | 5164 | 1.6 |
| C | Delay ~ peer-review + publication date + IUCN Red List category | 7 | -8422 | 5165 | 2.9 |
| D | Delay ~ 1 | 1 | -8521 | 5214 | 51.4 |
| E | Delay ~ publication date | 2 | -8521 | 5215 | 53.3 |
| F | Delay ~ peer-review | 2 | -8521 | 5216 | 53.3 |
| G | Delay ~ peer-review + publication date | 3 | -8521 | 5217 | 55.3 |
| **Mammals** | | | | | |
| Model rank | Model structure | df | logLik | qAIC | delta |
| Selected | Delay ~ peer-review + publication date + synopsis + IUCN Red List category | 9 | -9946 | 4700 | 0 |
| A | Delay ~ peer-review + synopsis + IUCN Red List category | 8 | -9990 | 4719 | 18.7 |
| B | Delay ~ publication date + synopsis + IUCN Red List category | 8 | -10043 | 4744 | 43.5 |
| C | Delay ~ synopsis + IUCN Red List category | 7 | -10099 | 4768 | 68 |
| D | Delay ~ peer-review + publication date + synopsis | 5 | -10166 | 4796 | 95.4 |
| E | Delay ~ peer-review + publication date + IUCN Red List category | 7 | -10196 | 4814 | 113.3 |
| F | Delay ~ peer-review + synopsis | 4 | -10223 | 4820 | 120 |
| G | Delay ~ peer-review + IUCN Red List category | 6 | -10236 | 4831 | 130.4 |
| H | Delay ~ publication date + synopsis | 4 | -10287 | 4851 | 150.2 |
| I | Delay ~ publication date + IUCN Red List category | 6 | -10291 | 4857 | 156.4 |
| J | Delay ~ IUCN Red List category | 5 | -10347 | 4881 | 180.3 |
| K | Delay ~ synopsis | 3 | -10359 | 4882 | 182.1 |
| L | Delay ~ peer-review + publication date | 3 | -10390 | 4897 | 196.9 |
| M | Delay ~ peer-review | 2 | -10422 | 4910 | 210 |
| N | Delay ~ publication date | 2 | -10501 | 4947 | 246.8 |
| O | Delay ~ 1 | 1 | -10545 | 4966 | 265.9 |
