# Supplementary material for: Reducing publication delay to improve the efficiency and impact of conservation science
Source: PeerJ. 2021 Oct 12;9:e12245. doi: 10.7717/peerj.12245 (PMC8519180; doi:10.7717/peerj.12245)
Supplement: Supplemental Information 12 — Significance level = 0.05. p-values of 0.000 represent p < 0.001. Comparisons were undertaken using the R package emmeans using the Tukey adjustment (Lenth, 2021, see main text). [file peerj-09-12245-s012.docx]

Table S9 — Results of pairwise comparisons of Estimated Marginal Means, derived from a quasi-Poisson Generalised Linear Model (see Methods), using the Tukey adjustment in the R package emmeans (Lenth 2021) to test for statistically significant differences between the publication delay of studies in different synopses. Significance level = 0.05. p-values of 0.000 represent p<0.001.

| Comparison | Estimate | Standard error | z-ratio | Adjusted p-value |
| --- | --- | --- | --- | --- |
| Bee Conservation - Bird Conservation | -0.686 | 0.099 | -6.938 | 0.000 |
| Bee Conservation - Farmland Conservation | -0.544 | 0.101 | -5.374 | 0.000 |
| Bee Conservation - Natural Pest Control | -0.711 | 0.119 | -5.985 | 0.000 |
| Bee Conservation - Control of Freshwater Invasive Species | -0.271 | 0.142 | -1.911 | 0.891 |
| Bee Conservation - Shrubland and Heathland Conservation | -0.668 | 0.115 | -5.826 | 0.000 |
| Bee Conservation - Terrestrial Mammal Conservation | -0.732 | 0.100 | -7.355 | 0.000 |
| Bee Conservation - Bat Conservation | -0.447 | 0.117 | -3.829 | 0.014 |
| Bee Conservation - Amphibian Conservation | -0.213 | 0.108 | -1.968 | 0.865 |
| Bee Conservation - Forest Conservation | -0.861 | 0.105 | -8.216 | 0.000 |
| Bee Conservation - Primate Conservation | -0.535 | 0.110 | -4.872 | 0.000 |
| Bee Conservation - Peatland Conservation | -0.719 | 0.110 | -6.518 | 0.000 |
| Bee Conservation - Mediterranean Farmland | -0.707 | 0.102 | -6.925 | 0.000 |
| Bee Conservation - Subtidal Benthic Invertebrate Conservation | -0.821 | 0.111 | -7.38 | 0.000 |
| Bee Conservation - Management of Captive Animals | -0.130 | 0.153 | -0.852 | 1.000 |
| Bee Conservation - Soil Fertility | -0.794 | 0.114 | -6.966 | 0.000 |
| Bee Conservation - Sustainable Aquaculture | -0.003 | 0.182 | -0.014 | 1.000 |
| Bird Conservation - Farmland Conservation | 0.142 | 0.040 | 3.593 | 0.032 |
| Bird Conservation - Natural Pest Control | -0.025 | 0.074 | -0.338 | 1.000 |
| Bird Conservation - Control of Freshwater Invasive Species | 0.414 | 0.107 | 3.866 | 0.012 |
| Bird Conservation - Shrubland and Heathland Conservation | 0.018 | 0.067 | 0.270 | 1.000 |
| Bird Conservation - Terrestrial Mammal Conservation | -0.047 | 0.035 | -1.324 | 0.997 |
| Bird Conservation - Bat Conservation | 0.238 | 0.071 | 3.373 | 0.065 |
| Bird Conservation - Amphibian Conservation | 0.473 | 0.055 | 8.544 | 0.000 |
| Bird Conservation - Forest Conservation | -0.175 | 0.048 | -3.642 | 0.027 |
| Bird Conservation - Primate Conservation | 0.150 | 0.058 | 2.577 | 0.443 |
| Bird Conservation - Peatland Conservation | -0.033 | 0.059 | -0.561 | 1.000 |
| Bird Conservation - Mediterranean Farmland | -0.021 | 0.042 | -0.503 | 1.000 |
| Bird Conservation - Subtidal Benthic Invertebrate Conservation | -0.135 | 0.061 | -2.214 | 0.718 |
| Bird Conservation - Management of Captive Animals | 0.556 | 0.121 | 4.590 | 0.001 |
| Bird Conservation - Soil Fertility | -0.108 | 0.066 | -1.642 | 0.970 |
| Bird Conservation - Sustainable Aquaculture | 0.683 | 0.156 | 4.373 | 0.001 |
| Farmland Conservation - Natural Pest Control | -0.167 | 0.077 | -2.179 | 0.741 |
| Farmland Conservation - Control of Freshwater Invasive Species | 0.273 | 0.109 | 2.504 | 0.498 |
| Farmland Conservation - Shrubland and Heathland Conservation | -0.124 | 0.070 | -1.772 | 0.94 |
| Farmland Conservation - Terrestrial Mammal Conservation | -0.189 | 0.041 | -4.627 | 0.000 |
| Farmland Conservation - Bat Conservation | 0.097 | 0.073 | 1.318 | 0.997 |
| Farmland Conservation - Amphibian Conservation | 0.331 | 0.059 | 5.634 | 0.000 |
| Farmland Conservation - Forest Conservation | -0.317 | 0.052 | -6.084 | 0.000 |
| Farmland Conservation - Primate Conservation | 0.008 | 0.062 | 0.136 | 1.000 |
| Farmland Conservation - Peatland Conservation | -0.175 | 0.062 | -2.813 | 0.283 |
| Farmland Conservation - Mediterranean Farmland | -0.163 | 0.046 | -3.525 | 0.04 |
| Farmland Conservation - Subtidal Benthic Invertebrate Conservation | -0.277 | 0.064 | -4.33 | 0.002 |
| Farmland Conservation - Management of Captive Animals | 0.414 | 0.123 | 3.369 | 0.066 |
| Farmland Conservation - Soil Fertility | -0.250 | 0.069 | -3.631 | 0.028 |
| Farmland Conservation - Sustainable Aquaculture | 0.541 | 0.158 | 3.436 | 0.054 |
| Natural Pest Control - Control of Freshwater Invasive Species | 0.439 | 0.126 | 3.501 | 0.044 |
| Natural Pest Control - Shrubland and Heathland Conservation | 0.043 | 0.094 | 0.458 | 1.000 |
| Natural Pest Control - Terrestrial Mammal Conservation | -0.022 | 0.074 | -0.293 | 1.000 |
| Natural Pest Control - Bat Conservation | 0.263 | 0.096 | 2.735 | 0.332 |
| Natural Pest Control - Amphibian Conservation | 0.497 | 0.086 | 5.808 | 0.000 |
| Natural Pest Control - Forest Conservation | -0.150 | 0.081 | -1.845 | 0.917 |
| Natural Pest Control - Primate Conservation | 0.175 | 0.088 | 1.996 | 0.851 |
| Natural Pest Control - Peatland Conservation | -0.008 | 0.088 | -0.095 | 1.000 |
| Natural Pest Control - Mediterranean Farmland | 0.004 | 0.078 | 0.048 | 1.000 |
| Natural Pest Control - Subtidal Benthic Invertebrate Conservation | -0.110 | 0.089 | -1.231 | 0.999 |
| Natural Pest Control - Management of Captive Animals | 0.581 | 0.138 | 4.216 | 0.003 |
| Natural Pest Control - Soil Fertility | -0.083 | 0.093 | -0.893 | 1.000 |
| Natural Pest Control - Sustainable Aquaculture | 0.708 | 0.169 | 4.179 | 0.003 |
| Control of Freshwater Invasive Species - Shrubland and Heathland Conservation | -0.396 | 0.121 | -3.264 | 0.091 |
| Control of Freshwater Invasive Species - Terrestrial Mammal Conservation | -0.461 | 0.107 | -4.297 | 0.002 |
| Control of Freshwater Invasive Species - Bat Conservation | -0.176 | 0.123 | -1.430 | 0.992 |
| Control of Freshwater Invasive Species - Amphibian Conservation | 0.058 | 0.115 | 0.505 | 1.000 |
| Control of Freshwater Invasive Species - Forest Conservation | -0.589 | 0.112 | -5.259 | 0.000 |
| Control of Freshwater Invasive Species - Primate Conservation | -0.264 | 0.117 | -2.258 | 0.686 |
| Control of Freshwater Invasive Species - Peatland Conservation | -0.448 | 0.117 | -3.828 | 0.014 |
| Control of Freshwater Invasive Species - Mediterranean Farmland | -0.436 | 0.109 | -3.986 | 0.008 |
| Control of Freshwater Invasive Species - Subtidal Benthic Invertebrate Conservation | -0.549 | 0.118 | -4.661 | 0.000 |
| Control of Freshwater Invasive Species - Management of Captive Animals | 0.141 | 0.158 | 0.894 | 1.000 |
| Control of Freshwater Invasive Species - Soil Fertility | -0.522 | 0.121 | -4.323 | 0.002 |
| Control of Freshwater Invasive Species - Sustainable Aquaculture | 0.269 | 0.186 | 1.444 | 0.992 |
| Shrubland and Heathland Conservation - Terrestrial Mammal Conservation | -0.065 | 0.067 | -0.962 | 1.000 |
| Shrubland and Heathland Conservation - Bat Conservation | 0.220 | 0.090 | 2.439 | 0.549 |
| Shrubland and Heathland Conservation - Amphibian Conservation | 0.455 | 0.080 | 5.700 | 0.000 |
| Shrubland and Heathland Conservation - Forest Conservation | -0.193 | 0.074 | -2.589 | 0.434 |
| Shrubland and Heathland Conservation - Primate Conservation | 0.132 | 0.082 | 1.616 | 0.974 |
| Shrubland and Heathland Conservation - Peatland Conservation | -0.051 | 0.082 | -0.624 | 1.000 |
| Shrubland and Heathland Conservation - Mediterranean Farmland | -0.039 | 0.070 | -0.557 | 1.000 |
| Shrubland and Heathland Conservation - Subtidal Benthic Invertebrate Conservation | -0.153 | 0.083 | -1.841 | 0.918 |
| Shrubland and Heathland Conservation - Management of Captive Animals | 0.538 | 0.134 | 4.016 | 0.007 |
| Shrubland and Heathland Conservation - Soil Fertility | -0.126 | 0.087 | -1.445 | 0.991 |
| Shrubland and Heathland Conservation - Sustainable Aquaculture | 0.665 | 0.166 | 4.000 | 0.007 |
| Terrestrial Mammal Conservation - Bat Conservation | 0.285 | 0.070 | 4.049 | 0.006 |
| Terrestrial Mammal Conservation - Amphibian Conservation | 0.519 | 0.056 | 9.289 | 0.000 |
| Terrestrial Mammal Conservation - Forest Conservation | -0.128 | 0.048 | -2.653 | 0.388 |
| Terrestrial Mammal Conservation - Primate Conservation | 0.197 | 0.059 | 3.340 | 0.072 |
| Terrestrial Mammal Conservation - Peatland Conservation | 0.013 | 0.059 | 0.226 | 1.000 |
| Terrestrial Mammal Conservation - Mediterranean Farmland | 0.026 | 0.042 | 0.613 | 1.000 |
| Terrestrial Mammal Conservation - Subtidal Benthic Invertebrate Conservation | -0.088 | 0.061 | -1.452 | 0.991 |
| Terrestrial Mammal Conservation - Management of Captive Animals | 0.602 | 0.121 | 4.967 | 0.000 |
| Terrestrial Mammal Conservation - Soil Fertility | -0.061 | 0.066 | -0.924 | 1.000 |
| Terrestrial Mammal Conservation - Sustainable Aquaculture | 0.730 | 0.156 | 4.669 | 0.000 |
| Bat Conservation - Amphibian Conservation | 0.234 | 0.082 | 2.841 | 0.266 |
| Bat Conservation - Forest Conservation | -0.413 | 0.077 | -5.356 | 0.000 |
| Bat Conservation - Primate Conservation | -0.088 | 0.085 | -1.042 | 1.000 |
| Bat Conservation - Peatland Conservation | -0.272 | 0.085 | -3.214 | 0.105 |
| Bat Conservation - Mediterranean Farmland | -0.260 | 0.073 | -3.561 | 0.036 |
| Bat Conservation - Subtidal Benthic Invertebrate Conservation | -0.373 | 0.085 | -4.381 | 0.001 |
| Bat Conservation - Management of Captive Animals | 0.317 | 0.135 | 2.342 | 0.623 |
| Bat Conservation - Soil Fertility | -0.346 | 0.089 | -3.869 | 0.012 |
| Bat Conservation - Sustainable Aquaculture | 0.445 | 0.168 | 2.655 | 0.387 |
| Amphibian Conservation - Forest Conservation | -0.647 | 0.065 | -10.026 | 0.000 |
| Amphibian Conservation - Primate Conservation | -0.322 | 0.073 | -4.431 | 0.001 |
| Amphibian Conservation - Peatland Conservation | -0.506 | 0.073 | -6.941 | 0.000 |
| Amphibian Conservation - Mediterranean Farmland | -0.494 | 0.060 | -8.248 | 0.000 |
| Amphibian Conservation - Subtidal Benthic Invertebrate Conservation | -0.607 | 0.074 | -8.169 | 0.000 |
| Amphibian Conservation - Management of Captive Animals | 0.083 | 0.129 | 0.646 | 1.000 |
| Amphibian Conservation - Soil Fertility | -0.580 | 0.079 | -7.373 | 0.000 |
| Amphibian Conservation - Sustainable Aquaculture | 0.211 | 0.162 | 1.299 | 0.997 |
| Forest Conservation - Primate Conservation | 0.325 | 0.067 | 4.840 | 0.000 |
| Forest Conservation - Peatland Conservation | 0.141 | 0.067 | 2.097 | 0.794 |
| Forest Conservation - Mediterranean Farmland | 0.154 | 0.052 | 2.938 | 0.214 |
| Forest Conservation - Subtidal Benthic Invertebrate Conservation | 0.040 | 0.068 | 0.583 | 1.000 |
| Forest Conservation - Management of Captive Animals | 0.730 | 0.125 | 5.824 | 0.000 |
| Forest Conservation - Soil Fertility | 0.067 | 0.073 | 0.914 | 1.000 |
| Forest Conservation - Sustainable Aquaculture | 0.858 | 0.16 | 5.378 | 0.000 |
| Primate Conservation - Peatland Conservation | -0.184 | 0.075 | -2.432 | 0.554 |
| Primate Conservation - Mediterranean Farmland | -0.171 | 0.063 | -2.736 | 0.331 |
| Primate Conservation - Subtidal Benthic Invertebrate Conservation | -0.285 | 0.077 | -3.721 | 0.02 |
| Primate Conservation - Management of Captive Animals | 0.405 | 0.130 | 3.119 | 0.136 |
| Primate Conservation - Soil Fertility | -0.258 | 0.081 | -3.192 | 0.111 |
| Primate Conservation - Sustainable Aquaculture | 0.533 | 0.163 | 3.266 | 0.09 |
| Peatland Conservation - Mediterranean Farmland | 0.012 | 0.063 | 0.193 | 1.000 |
| Peatland Conservation - Subtidal Benthic Invertebrate Conservation | -0.102 | 0.077 | -1.324 | 0.997 |
| Peatland Conservation - Management of Captive Animals | 0.589 | 0.130 | 4.526 | 0.001 |
| Peatland Conservation - Soil Fertility | -0.074 | 0.081 | -0.917 | 1.000 |
| Peatland Conservation - Sustainable Aquaculture | 0.716 | 0.163 | 4.388 | 0.001 |
| Mediterranean Farmland - Subtidal Benthic Invertebrate Conservation | -0.114 | 0.064 | -1.787 | 0.936 |
| Mediterranean Farmland - Management of Captive Animals | 0.577 | 0.123 | 4.689 | 0.000 |
| Mediterranean Farmland - Soil Fertility | -0.087 | 0.069 | -1.252 | 0.998 |
| Mediterranean Farmland - Sustainable Aquaculture | 0.704 | 0.158 | 4.469 | 0.001 |
| Subtidal Benthic Invertebrate Conservation - Management of Captive Animals | 0.691 | 0.131 | 5.283 | 0.000 |
| Subtidal Benthic Invertebrate Conservation - Soil Fertility | 0.027 | 0.082 | 0.331 | 1.000 |
| Subtidal Benthic Invertebrate Conservation - Sustainable Aquaculture | 0.818 | 0.164 | 4.998 | 0.000 |
| Management of Captive Animals - Soil Fertility | -0.663 | 0.133 | -4.978 | 0.000 |
| Management of Captive Animals - Sustainable Aquaculture | 0.128 | 0.195 | 0.656 | 1.000 |
| Soil Fertility - Sustainable Aquaculture | 0.791 | 0.166 | 4.771 | 0.000 |
