# Supplementary material for: Reducing publication delay to improve the efficiency and impact of conservation science
Source: PeerJ. 2021 Oct 12;9:e12245. doi: 10.7717/peerj.12245 (PMC8519180; doi:10.7717/peerj.12245)
Supplement: Supplemental Information 13 — Estimate is the log odds difference between categories. Significance level = 0.05. p-values of 0.000 represent p < 0.001. Comparisons were undertaken using the R package emmeans using the Tukey adjustment (Lenth, 2021, see main text). [file peerj-09-12245-s013.docx]

Table S10 — Results of pairwise comparisons of Estimated Marginal Means, derived from a quasi-Poisson Generalised Linear Model (see Methods), using the Tukey adjustment in the R package emmeans (Lenth 2021) to test for statistically significant differences between the publication delay of studies from different publication sources. Estimate is the log odds difference between categories. Significance level = 0.05. p-values of 0.000 represent p<0.001.

| Comparison | Estimate | Standard error | z-ratio | Adjusted p-value |
| --- | --- | --- | --- | --- |
| Non-peer-reviewed -  Peer-reviewed | -0.441 | 0.065 | -6.779 | 0.000 |
| Category | Estimated Marginal Mean | Standard error | Lower 95% confidence interval | Upper 95% confidence interval |
| Non-peer-reviewed | 1.901 | 0.124 | 1.643 | 2.200 |
| Peer-reviewed | 2.954 | 0.053 | 2.837 | 3.074 |
