# Supplementary material for: Reducing publication delay to improve the efficiency and impact of conservation science
Source: PeerJ. 2021 Oct 12;9:e12245. doi: 10.7717/peerj.12245 (PMC8519180; doi:10.7717/peerj.12245)
Supplement: Supplemental Information 14 — p-values of 0.000 represent p < 0.001. [file peerj-09-12245-s014.docx]

Table S11 — Results of quasi-Poisson Generalised Linear Model (GLM) (see Methods) for the main taxonomic analyses. p-values of 0.000 represent p<0.001.

| Amphibians, birds, and mammals | | | | |
| --- | --- | --- | --- | --- |
| Parameter | Estimate | Standard Error | t-value | p-value |
| Intercept | -11.604 | 2.755 | -4.212 | 0 |
| Publication Date | 0.006 | 0.001 | 4.445 | 0 |
| Terrestrial Mammal Conservation | 0.132 | 0.029 | 4.582 | 0 |
| Bat Conservation | -0.281 | 0.049 | -5.716 | 0 |
| Amphibian Conservation | -0.492 | 0.053 | -9.214 | 0 |
| Primate Conservation | -0.315 | 0.064 | -4.942 | 0 |
| Peer-reviewed (yes) | 0.532 | 0.085 | 6.293 | 0 |
| Near Threatened | -0.025 | 0.039 | -0.641 | 0.522 |
| Vulnerable | -0.058 | 0.04 | -1.448 | 0.148 |
| Endangered | 0.427 | 0.036 | 11.926 | 0 |
| Critically Endangered | 0.189 | 0.061 | 3.126 | 0.002 |
| Amphibians only | | | | |
| Parameter | Estimate | Standard Error | t-value | p-value |
| Intercept | -31.018 | 12.398 | -2.502 | 0.013 |
| Publication Date | 0.016 | 0.006 | 2.533 | 0.012 |
| Peer-reviewed (yes) | 0.334 | 0.128 | 2.607 | 0.009 |
| Near Threatened | 0.221 | 0.209 | 1.057 | 0.291 |
| Vulnerable | 0.024 | 0.136 | 0.18 | 0.857 |
| Endangered | 0.024 | 0.149 | 0.164 | 0.87 |
| Critically Endangered | 0.079 | 0.156 | 0.507 | 0.612 |
| Birds only | | | | |
| Parameter | Estimate | Standard Error | t-value | p-value |
| Intercept | 1.199 | 0.021 | 56.252 | 0 |
| Near Threatened | -0.027 | 0.046 | -0.596 | 0.551 |
| Vulnerable | -0.243 | 0.071 | -3.431 | 0.001 |
| Endangered | 0.487 | 0.073 | 6.636 | 0 |
| Critically Endangered | -0.247 | 0.135 | -1.828 | 0.068 |
| Mammals only | | | | |
| Parameter | Estimate | Standard Error | t-value | p-value |
| Intercept | -18.86 | 4.319 | -4.367 | 0 |
| Publication Date | 0.01 | 0.002 | 4.461 | 0 |
| Peer-reviewed (yes) | 0.885 | 0.152 | 5.829 | 0 |
| Near Threatened | -0.021 | 0.073 | -0.284 | 0.776 |
| Vulnerable | 0.027 | 0.054 | 0.49 | 0.624 |
| Endangered | 0.453 | 0.046 | 9.847 | 0 |
| Critically Endangered | 0.33 | 0.081 | 4.081 | 0 |
| Bat Conservation | -0.431 | 0.052 | -8.26 | 0 |
| Primate Conservation | -0.488 | 0.071 | -6.872 | 0 |
