# Supplementary material for: Reducing publication delay to improve the efficiency and impact of conservation science
Source: PeerJ. 2021 Oct 12;9:e12245. doi: 10.7717/peerj.12245 (PMC8519180; doi:10.7717/peerj.12245)
Supplement: Supplemental Information 15 — Estimate is the log odds difference between categories. Significance level = 0.05. p-values of 0.000 represent p < 0.001. Comparisons were undertaken using the R package emmeans using the Tukey adjustment (Lenth, 2021, see main text) and Estimated Marginal Means were derived from a quasi–Poisson Generalised Linear Model (see Methods). [file peerj-09-12245-s015.docx]

Table S12 — Results of pairwise comparisons of Estimated Marginal Means, derived from a quasi-Poisson Generalised Linear Model (see Methods), using the Tukey adjustment in the R package emmeans (Lenth 2021) to test for statistically significant differences between the publication delay of studies on species with different IUCN Red List statuses. Estimate is the log odds difference between categories. Significance level = 0.05. p-values of 0.000 represent p<0.001.

| Amphibians, birds, and mammals | | | | |
| --- | --- | --- | --- | --- |
| Comparison | Estimate | Standard error | z-ratio | Adjusted p-value |
| LC - NT | 0.025 | 0.039 | 0.641 | 0.968 |
| LC - VU | 0.058 | 0.040 | 1.448 | 0.597 |
| LC - EN | -0.427 | 0.036 | -11.926 | 0.000 |
| LC - CR | -0.189 | 0.061 | -3.126 | 0.015 |
| NT - VU | 0.033 | 0.051 | 0.645 | 0.968 |
| NT - EN | -0.452 | 0.048 | -9.353 | 0.000 |
| NT - CR | -0.214 | 0.069 | -3.125 | 0.015 |
| VU - EN | -0.485 | 0.047 | -10.24 | 0.000 |
| VU - CR | -0.247 | 0.068 | -3.618 | 0.003 |
| EN - CR | 0.238 | 0.063 | 3.795 | 0.001 |
| Amphibians only | | | | |
| Comparison | Estimate | Standard error | z-ratio | Adjusted p-value |
| LC - NT | -0.221 | 0.209 | -1.057 | 0.829 |
| LC - VU | -0.024 | 0.136 | -0.180 | 1.000 |
| LC - EN | -0.024 | 0.149 | -0.164 | 1.000 |
| LC - CR | -0.079 | 0.156 | -0.507 | 0.987 |
| NT - VU | 0.196 | 0.239 | 0.820 | 0.924 |
| NT - EN | 0.196 | 0.245 | 0.800 | 0.931 |
| NT - CR | 0.141 | 0.250 | 0.564 | 0.980 |
| VU - EN | 0.000 | 0.188 | 0.001 | 1.000 |
| VU - CR | -0.055 | 0.196 | -0.280 | 0.999 |
| EN - CR | -0.055 | 0.202 | -0.271 | 0.999 |
| Birds only | | | | |
| Comparison | Estimate | Standard error | z-ratio | Adjusted p-value |
| LC - NT | 0.027 | 0.046 | 0.596 | 0.976 |
| LC - VU | 0.243 | 0.071 | 3.431 | 0.005 |
| LC - EN | -0.487 | 0.073 | -6.636 | 0.000 |
| LC - CR | 0.247 | 0.135 | 1.828 | 0.358 |
| NT - VU | 0.215 | 0.079 | 2.728 | 0.050 |
| NT - EN | -0.514 | 0.081 | -6.331 | 0.000 |
| NT - CR | 0.219 | 0.140 | 1.573 | 0.515 |
| VU - EN | -0.729 | 0.097 | -7.495 | 0.000 |
| VU - CR | 0.004 | 0.149 | 0.029 | 1.000 |
| EN - CR | 0.734 | 0.151 | 4.868 | 0.000 |
| Mammals only | | | | |
| Comparison | Estimate | Standard error | z-ratio | Adjusted p-value |
| LC - NT | 0.021 | 0.073 | 0.284 | 0.999 |
| LC - VU | -0.027 | 0.054 | -0.490 | 0.988 |
| LC - EN | -0.453 | 0.046 | -9.847 | 0.000 |
| LC - CR | -0.330 | 0.081 | -4.081 | 0.000 |
| NT - VU | -0.047 | 0.081 | -0.583 | 0.978 |
| NT - EN | -0.474 | 0.077 | -6.142 | 0.000 |
| NT - CR | -0.351 | 0.102 | -3.442 | 0.005 |
| VU - EN | -0.426 | 0.060 | -7.151 | 0.000 |
| VU - CR | -0.304 | 0.089 | -3.407 | 0.006 |
| EN - CR | 0.123 | 0.079 | 1.551 | 0.529 |
