# Supplementary material for: Reducing publication delay to improve the efficiency and impact of conservation science
Source: PeerJ. 2021 Oct 12;9:e12245. doi: 10.7717/peerj.12245 (PMC8519180; doi:10.7717/peerj.12245)
Supplement: Supplemental Information 17 — These include journals that have previously published tests of conservation interventions (based on our study’s dataset), plus those who name includes the terms ‘wildlife’, ‘environment’, ‘biology’, ‘ecology’, ‘wildlife’, and ‘zoology’. These data were generated using code available here: http://doi.org/10.5281/zenodo.45516 (Himmelstein & Powell, 2016). Delay type = ‘Acceptance’ refers to number of days from submission to acceptance, delay type = ‘Publication’ refers to the number of days from acceptance to online publication. We caution, however, that this data is based on studies for which data on delays could be extracted and may not be representative of delays at each journal, particularly where data was only available for a very small number of studies (see ‘Number of studies’ column) (Himmelstein, 2016; Himmelstein & Powell, 2016). [file peerj-09-12245-s017.docx]

Table S14 – Median and mean delays (in days) of studies published in different journals from 2015-2021 that may be relevant to conservation scientists – these include journals that have previously published tests of conservation interventions (based on our study’s dataset), plus those who name includes the terms ‘wildlife’, ‘environment’, ‘biology’, ‘ecology’, ‘wildlife’, and ‘zoology’. These data were generated using code available here: <http://doi.org/10.5281/zenodo.45516> (Himmelstein & Powell, 2016). Delay type = ‘Acceptance’ refers to number of days from submission to acceptance, delay type = ‘Publication’ refers to the number of days from acceptance to online publication.

We caution, however, that **this data is based on studies for which data on delays could be extracted and may not be representative of delays at each journal, particularly where data was only available for a very small number of studies (see ‘Number of studies’ column)** (Himmelstein 2016; Himmelstein & Powell 2016).

| Journal | Delay type | Number of studies | Median delay | Mean delay |
| --- | --- | --- | --- | --- |
| Accident; analysis and prevention | Acceptance | 2979 | 170 | 199.432 |
| Addiction biology | Acceptance | 56 | 129.5 | 137.375 |
| Advances in enzyme regulation | Acceptance | 25 | 3 | 3 |
| Agriculture, ecosystems & environment | Acceptance | 1 | 139 | 139 |
| Ambio | Acceptance | 299 | 173 | 184.274 |
| American journal of botany | Acceptance | 138 | 106 | 115.783 |
| American journal of primatology | Acceptance | 606 | 137.5 | 157.51 |
| Amphibian & reptile conservation | Acceptance | 2 | 92 | 92 |
| Animal behaviour | Acceptance | 32 | 124.5 | 136.375 |
| Animal conservation | Acceptance | 1 | 274 | 274 |
| Animal: an international journal of animal bioscience | Acceptance | 1 | 189 | 189 |
| Annals of botany | Acceptance | 117 | 88 | 99.735 |
| Annals of clinical microbiology and antimicrobials | Acceptance | 410 | 86.5 | 94.8293 |
| Annals of occupational and environmental medicine | Acceptance | 118 | 132.5 | 149.407 |
| Apidologie | Acceptance | 1 | 130 | 130 |
| Applied and environmental microbiology | Acceptance | 651 | 58 | 60.9278 |
| Applied entomology and zoology | Acceptance | 1 | 41 | 41 |
| Applied microbiology and biotechnology | Acceptance | 8030 | 64 | 72.8346 |
| Applied soil ecology : a section of Agriculture, Ecosystems & Environment | Acceptance | 2 | 146.5 | 146.5 |
| Aquaculture (Amsterdam, Netherlands) | Acceptance | 2 | 63.5 | 63.5 |
| Aquatic conservation : marine and freshwater ecosystems | Acceptance | 1 | 160 | 160 |
| Aquatic ecology | Acceptance | 1 | 118 | 118 |
| Archives of environmental contamination and toxicology | Acceptance | 1717 | 129 | 144.63 |
| Archives of oral biology | Acceptance | 1918 | 137 | 152.513 |
| Atmospheric environment (Oxford, England : 1994) | Acceptance | 3 | 123 | 174 |
| Behavioral ecology : official journal of the International Society for Behavioral Ecology | Acceptance | 62 | 145.5 | 167.694 |
| Behavioral ecology and sociobiology | Acceptance | 98 | 111.5 | 130.194 |
| Biochemistry and molecular biology education : a bimonthly publication of the International Union of Biochemistry and Molecular Biology | Acceptance | 200 | 73.5 | 90.465 |
| Biodiversity and conservation | Acceptance | 6 | 142 | 147.833 |
| BioEssays : news and reviews in molecular, cellular and developmental biology | Acceptance | 29 | 74 | 80.2069 |
| Bioinformatics and biology insights | Acceptance | 46 | 65 | 65.3261 |
| Biological conservation | Acceptance | 6 | 161 | 144.667 |
| Biological invasions | Acceptance | 5 | 190 | 213.4 |
| Biology & philosophy | Acceptance | 24 | 202.5 | 193.708 |
| Biology and fertility of soils | Acceptance | 2 | 94.5 | 94.5 |
| Biology direct | Acceptance | 481 | 63 | 73.817 |
| Biology letters | Acceptance | 2 | 112 | 112 |
| Biology of reproduction | Acceptance | 186 | 121.5 | 128.548 |
| Biology of the neonate | Acceptance | 234 | 146 | 156.594 |
| Biology open | Acceptance | 205 | 48 | 60.9366 |
| Biomass & bioenergy | Acceptance | 5 | 266 | 241.4 |
| Biomedical and environmental sciences : BES | Acceptance | 506 | 120.5 | 134.502 |
| Biomedical engineering and computational biology | Acceptance | 2 | 42 | 42 |
| Bioresource technology | Acceptance | 13089 | 75 | 111.651 |
| BMC biology | Acceptance | 820 | 78.5 | 87.5841 |
| BMC chemical biology | Acceptance | 39 | 156 | 176.974 |
| BMC developmental biology | Acceptance | 830 | 151 | 156.976 |
| BMC ecology | Acceptance | 307 | 144 | 160.107 |
| BMC evolutionary biology | Acceptance | 2943 | 143 | 154.772 |
| BMC molecular biology | Acceptance | 712 | 147 | 157.09 |
| BMC plant biology | Acceptance | 2034 | 133 | 146.58 |
| BMC structural biology | Acceptance | 481 | 137 | 146.528 |
| BMC systems biology | Acceptance | 1173 | 158 | 174.972 |
| Brazilian journal of biology = Revista brasleira de biologia | Acceptance | 1302 | 119 | 168.035 |
| Brazilian journal of microbiology : [publication of the Brazilian Society for Microbiology] | Acceptance | 1345 | 297 | 323.917 |
| Bulletin of environmental contamination and toxicology | Acceptance | 3050 | 155 | 178.875 |
| Bulletin of mathematical biology | Acceptance | 1185 | 208 | 229.184 |
| Cell biology and toxicology | Acceptance | 393 | 117 | 134.412 |
| Cell biology education | Acceptance | 88 | 81 | 94.3864 |
| Cell biology international | Acceptance | 1427 | 133 | 147.601 |
| Cellular & molecular biology letters | Acceptance | 451 | 136 | 138.987 |
| Cellular and molecular biology (Noisy-le-Grand, France) | Acceptance | 663 | 54 | 91.6094 |
| Chembiochem : a European journal of chemical biology | Acceptance | 42 | 41 | 41.7857 |
| Chemistry & biology | Acceptance | 1612 | 88 | 107.853 |
| Clinical microbiology and infection : the official publication of the European Society of Clinical Microbiology and Infectious Diseases | Acceptance | 854 | 91.5 | 98.6253 |
| Communicative & integrative biology | Acceptance | 457 | 3 | 14.4726 |
| Comparative biochemistry and physiology. Part A, Molecular & integrative physiology | Acceptance | 2354 | 94 | 111.236 |
| Comparative biochemistry and physiology. Part B, Biochemistry & molecular biology | Acceptance | 1687 | 93 | 107.437 |
| Comparative immunology, microbiology and infectious diseases | Acceptance | 306 | 127 | 142.359 |
| Computational biology and chemistry | Acceptance | 644 | 113 | 129.646 |
| Conservation biology : the journal of the Society for Conservation Biology | Acceptance | 581 | 163 | 188.492 |
| Conservation genetics (Print) | Acceptance | 1 | 40 | 40 |
| Conservation genetics resources | Acceptance | 3 | 26 | 40.3333 |
| Cryobiology | Acceptance | 1081 | 122 | 146.132 |
| Current biology : CB | Acceptance | 4058 | 83.5 | 102.53 |
| Current chemical biology | Acceptance | 1 | 27 | 27 |
| Current issues in molecular biology | Acceptance | 10 | 70 | 63.9 |
| Current opinion in chemical biology | Acceptance | 1013 | 54 | 57.617 |
| Current opinion in environmental sustainability | Acceptance | 3 | 119 | 115 |
| Current opinion in plant biology | Acceptance | 1060 | 65 | 66.7396 |
| Current opinion in structural biology | Acceptance | 1014 | 57 | 60.4665 |
| Developmental biology | Acceptance | 5190 | 124 | 139.111 |
| Diagnostic microbiology and infectious disease | Acceptance | 2617 | 94 | 104.971 |
| EcoHealth | Acceptance | 428 | 183 | 215.311 |
| Ecological economics : the journal of the International Society for Ecological Economics | Acceptance | 6 | 179.5 | 367.833 |
| Ecological engineering | Acceptance | 1 | 138 | 138 |
| Ecological indicators | Acceptance | 3 | 150 | 146.333 |
| Ecology and evolution | Acceptance | 1530 | 70 | 82.0157 |
| Ecology letters | Acceptance | 453 | 91 | 95.0773 |
| Economics and human biology | Acceptance | 360 | 222 | 230.936 |
| Ecosystems (New York, N.Y.) | Acceptance | 2 | 143.5 | 143.5 |
| Ecotoxicology and environmental safety | Acceptance | 3327 | 136 | 168.6 |
| Energy & environmental science | Acceptance | 2 | 78 | 78 |
| Environment international | Acceptance | 1776 | 119 | 133.278 |
| Environmental and experimental botany | Acceptance | 3 | 103 | 102.333 |
| Environmental and molecular mutagenesis | Acceptance | 355 | 93 | 118.639 |
| Environmental biology of fishes | Acceptance | 1 | 158 | 158 |
| Environmental biosafety research | Acceptance | 22 | 156.5 | 174.318 |
| Environmental chemistry letters | Acceptance | 6 | 101.5 | 104.167 |
| Environmental engineering science | Acceptance | 75 | 163 | 194.56 |
| Environmental entomology | Acceptance | 173 | 136 | 163.393 |
| Environmental geochemistry and health | Acceptance | 914 | 183 | 221.186 |
| Environmental health : a global access science source | Acceptance | 847 | 141 | 155.124 |
| Environmental health and preventive medicine | Acceptance | 791 | 109 | 135.426 |
| Environmental health and toxicology | Acceptance | 90 | 75.5 | 87.9889 |
| Environmental health insights | Acceptance | 33 | 54 | 67.7273 |
| Environmental health perspectives | Acceptance | 2250 | 185 | 202.683 |
| Environmental management | Acceptance | 1659 | 301 | 324.409 |
| Environmental microbiology | Acceptance | 920 | 98 | 120.463 |
| Environmental microbiology reports | Acceptance | 270 | 103 | 136.037 |
| Environmental modelling & software : with environment data news | Acceptance | 2 | 205 | 205 |
| Environmental monitoring and assessment | Acceptance | 5999 | 180 | 195.891 |
| Environmental pollution (Barking, Essex : 1987) | Acceptance | 6961 | 121 | 137.966 |
| Environmental research | Acceptance | 2060 | 148 | 163.683 |
| Environmental science & technology letters | Acceptance | 4 | 23.5 | 22 |
| Environmental science and pollution research international | Acceptance | 5837 | 103 | 118.162 |
| Environmental toxicology | Acceptance | 680 | 132.5 | 147.676 |
| Environmental toxicology and chemistry / SETAC | Acceptance | 2459 | 120 | 128.511 |
| Environmental toxicology and pharmacology | Acceptance | 2021 | 129 | 140.157 |
| Environmetrics | Acceptance | 5 | 267 | 219.8 |
| Estuarine, coastal and shelf science | Acceptance | 1 | 262 | 262 |
| Ethology, ecology & evolution | Acceptance | 1 | 120 | 120 |
| European journal of clinical microbiology & infectious diseases : official publication of the European Society of Clinical Microbiology | Acceptance | 2051 | 80 | 89.5817 |
| European journal of microbiology & immunology | Acceptance | 143 | 7 | 12.9161 |
| European journal of soil biology | Acceptance | 3 | 107 | 109 |
| European journal of soil science | Acceptance | 2 | 220 | 220 |
| Evolutionary biology | Acceptance | 20 | 106.5 | 115 |
| Evolutionary ecology | Acceptance | 1 | 167 | 167 |
| Experimental biology and medicine (Maywood, N.J.) | Acceptance | 130 | 104.5 | 108.638 |
| FASEB journal : official publication of the Federation of American Societies for Experimental Biology | Acceptance | 431 | 95 | 103.608 |
| FEMS microbiology ecology | Acceptance | 862 | 118 | 129.386 |
| FEMS microbiology letters | Acceptance | 1665 | 69 | 77.8655 |
| Field crops research | Acceptance | 2 | 199.5 | 199.5 |
| Folia primatologica; international journal of primatology | Acceptance | 268 | 186.5 | 206.284 |
| Food and environmental virology | Acceptance | 151 | 101 | 109.437 |
| Forest ecology and management | Acceptance | 4 | 93.5 | 105 |
| Frontiers in zoology | Acceptance | 399 | 102 | 115.163 |
| Functional ecology | Acceptance | 2 | 166 | 166 |
| Fungal biology | Acceptance | 623 | 149 | 166.266 |
| Fungal biology reviews | Acceptance | 1 | 183 | 183 |
| Fungal ecology | Acceptance | 1 | 162 | 162 |
| Fungal genetics and biology : FG & B | Acceptance | 1358 | 98.5 | 113.08 |
| Genesis (New York, N.Y. : 2000) | Acceptance | 366 | 71.5 | 98.7923 |
| Genetics and molecular biology | Acceptance | 681 | 140 | 150.944 |
| Geoderma | Acceptance | 3 | 208 | 193 |
| Global change biology | Acceptance | 1153 | 99 | 112.432 |
| Global change biology. Bioenergy | Acceptance | 1 | 35 | 35 |
| Global environmental change : human and policy dimensions | Acceptance | 3 | 149 | 161.667 |
| Historical biology | Acceptance | 2 | 47 | 47 |
| Hormones and behavior | Acceptance | 1914 | 115 | 125.387 |
| Hydrobiologia | Acceptance | 2 | 169 | 169 |
| In vitro cellular & developmental biology. Animal | Acceptance | 847 | 94 | 106.419 |
| Infection ecology & epidemiology | Acceptance | 103 | 113 | 120.767 |
| Insect biochemistry and molecular biology | Acceptance | 1319 | 75 | 94.5891 |
| Insect molecular biology | Acceptance | 10 | 154 | 152.4 |
| Insect science | Acceptance | 1 | 116 | 116 |
| Insectes sociaux | Acceptance | 14 | 168 | 168.714 |
| Integrated environmental assessment and management | Acceptance | 403 | 144 | 156.722 |
| International archives of occupational and environmental health | Acceptance | 1325 | 181 | 198.328 |
| International biodeterioration & biodegradation | Acceptance | 2 | 172.5 | 172.5 |
| International journal for parasitology. Parasites and wildlife | Acceptance | 126 | 65.5 | 83.6984 |
| International journal of biochemistry and molecular biology | Acceptance | 89 | 23 | 28.3596 |
| International journal of environmental analytical chemistry | Acceptance | 1 | 90 | 90 |
| International journal of environmental research and public health | Acceptance | 3196 | 54 | 61.8676 |
| International journal of evolutionary biology | Acceptance | 142 | 86 | 90.162 |
| International journal of hygiene and environmental health | Acceptance | 746 | 150.5 | 171.735 |
| International journal of medical microbiology : IJMM | Acceptance | 557 | 123 | 149.102 |
| International journal of occupational medicine and environmental health | Acceptance | 286 | 138.5 | 168.503 |
| International journal of primatology | Acceptance | 47 | 123 | 150.106 |
| International microbiology : the official journal of the Spanish Society for Microbiology | Acceptance | 79 | 65 | 77.7215 |
| Iranian journal of environmental health science & engineering | Acceptance | 51 | 10 | 51.4902 |
| Journal of agricultural, biological, and environmental statistics | Acceptance | 2 | 137 | 137 |
| Journal of bioinformatics and computational biology | Acceptance | 498 | 110 | 126.201 |
| Journal of biology | Acceptance | 43 | 117 | 141.744 |
| Journal of chemical biology | Acceptance | 80 | 72 | 79.05 |
| Journal of chemical ecology | Acceptance | 3754 | 111 | 131.425 |
| Journal of comparative physiology. B, Biochemical, systemic, and environmental physiology | Acceptance | 932 | 111 | 122.891 |
| Journal of economic entomology | Acceptance | 328 | 115.5 | 153.058 |
| Journal of environmental and public health | Acceptance | 293 | 99 | 111.096 |
| Journal of environmental health science & engineering | Acceptance | 276 | 252.5 | 291.203 |
| Journal of environmental management | Acceptance | 4309 | 241 | 277.479 |
| Journal of environmental radioactivity | Acceptance | 2108 | 155 | 188.744 |
| Journal of environmental sciences (China) | Acceptance | 518 | 96 | 102.048 |
| Journal of ethnobiology and ethnomedicine | Acceptance | 539 | 126 | 143.135 |
| Journal of evolutionary biology | Acceptance | 833 | 105 | 131.138 |
| Journal of experimental marine biology and ecology | Acceptance | 2 | 110 | 110 |
| Journal of experimental zoology. Part A, Ecological genetics and physiology | Acceptance | 316 | 122 | 129.339 |
| Journal of experimental zoology. Part B, Molecular and developmental evolution | Acceptance | 234 | 123 | 137.376 |
| Journal of exposure analysis and environmental epidemiology | Acceptance | 18 | 151 | 257.278 |
| Journal of exposure science & environmental epidemiology | Acceptance | 265 | 141 | 154.491 |
| Journal of fish biology | Acceptance | 738 | 172 | 199.075 |
| Journal of fish diseases | Acceptance | 456 | 72 | 81.682 |
| Journal of industrial microbiology & biotechnology | Acceptance | 2137 | 101 | 121.796 |
| Journal of insect science (Online) | Acceptance | 270 | 117.5 | 161.496 |
| Journal of integrative plant biology | Acceptance | 291 | 74 | 80.9588 |
| Journal of invertebrate pathology | Acceptance | 1411 | 108 | 121.58 |
| Journal of mammary gland biology and neoplasia | Acceptance | 230 | 38 | 45.4043 |
| Journal of mathematical biology | Acceptance | 3 | 187 | 149 |
| Journal of microbiology (Seoul, Korea) | Acceptance | 1005 | 81 | 94.1254 |
| Journal of microbiology and biotechnology | Acceptance | 95 | 70 | 76.1684 |
| Journal of molecular biology | Acceptance | 8528 | 77 | 85.3847 |
| Journal of molecular cell biology | Acceptance | 49 | 115 | 113.102 |
| Journal of molecular microbiology and biotechnology | Acceptance | 9 | 134 | 175.889 |
| Journal of pest science | Acceptance | 17 | 135 | 154.706 |
| Journal of pharmacological and toxicological methods | Acceptance | 749 | 68 | 88.0587 |
| Journal of pharmacy & bioallied sciences | Acceptance | 540 | 56 | 66.6148 |
| Journal of photochemistry and photobiology. B, Biology | Acceptance | 1828 | 101 | 115.058 |
| Journal of soils and sediments | Acceptance | 1 | 155 | 155 |
| Journal of structural biology | Acceptance | 1839 | 92 | 102.141 |
| Journal of theoretical biology | Acceptance | 4656 | 151 | 166.876 |
| Journal of thermal biology | Acceptance | 222 | 116.5 | 129.676 |
| Journal of trace elements in medicine and biology : organ of the Society for Minerals and Trace Elements (GMS) | Acceptance | 611 | 129 | 165.409 |
| Journal of vector ecology : journal of the Society for Vector Ecology | Acceptance | 121 | 72 | 88.6116 |
| Journal of zoology (London, England : 1987) | Acceptance | 5 | 98 | 110.8 |
| Kinetoplastid biology and disease | Acceptance | 46 | 97 | 100.935 |
| Landscape and urban planning | Acceptance | 1 | 242 | 242 |
| Landscape ecology | Acceptance | 4 | 155 | 129.75 |
| Marine biology | Acceptance | 77 | 168 | 177.584 |
| Marine environmental research | Acceptance | 1047 | 114 | 171.006 |
| Marine pollution bulletin | Acceptance | 3791 | 105 | 124.386 |
| Mathematical medicine and biology : a journal of the IMA | Acceptance | 17 | 209 | 239.706 |
| Matrix biology : journal of the International Society for Matrix Biology | Acceptance | 742 | 96 | 114.257 |
| Medical and veterinary entomology | Acceptance | 148 | 112 | 139.797 |
| Medical microbiology and immunology | Acceptance | 218 | 89 | 90.2294 |
| Medical mycology case reports | Acceptance | 148 | 36.5 | 44.2838 |
| Methods in ecology and evolution / British Ecological Society | Acceptance | 15 | 99 | 125.8 |
| Microbial ecology | Acceptance | 2110 | 110 | 122.849 |
| Microbial ecology in health and disease | Acceptance | 38 | 110.5 | 124.763 |
| Microbiology (Reading, England) | Acceptance | 42 | 98.5 | 89.2857 |
| Microbiology and immunology | Acceptance | 300 | 83.5 | 97.1433 |
| Microbiology insights | Acceptance | 13 | 50 | 50 |
| Molecular and cellular biology | Acceptance | 226 | 88 | 94.031 |
| Molecular biology and evolution | Acceptance | 4 | 141 | 140 |
| Molecular biology international | Acceptance | 91 | 63 | 76.8022 |
| Molecular biology of the cell | Acceptance | 268 | 118.5 | 138.843 |
| Molecular biology reports | Acceptance | 4680 | 157 | 179.109 |
| Molecular ecology | Acceptance | 1441 | 117 | 169.883 |
| Molecular ecology notes | Acceptance | 4 | 75.5 | 71.75 |
| Molecular ecology resources | Acceptance | 460 | 105 | 146.522 |
| Molecular systems biology | Acceptance | 581 | 129 | 140.491 |
| Molekuliarnaia biologiia | Acceptance | 71 | 44 | 51.0141 |
| Movement ecology | Acceptance | 65 | 121 | 140.015 |
| Mutation research. Genetic toxicology and environmental mutagenesis | Acceptance | 258 | 87.5 | 116.298 |
| Mycorrhiza | Acceptance | 812 | 127 | 141.037 |
| Nature | Acceptance | 11006 | 111 | 135.885 |
| Nature chemical biology | Acceptance | 968 | 115 | 129.157 |
| Nature structural & molecular biology | Acceptance | 1917 | 102 | 116.795 |
| Nature structural biology | Acceptance | 162 | 96 | 96.821 |
| Neurobiology of disease | Acceptance | 2850 | 108 | 120.55 |
| Neurobiology of learning and memory | Acceptance | 1420 | 101 | 117.856 |
| Nitric oxide : biology and chemistry / official journal of the Nitric Oxide Society | Acceptance | 856 | 115 | 126.794 |
| Occupational and environmental medicine | Acceptance | 107 | 161 | 162.813 |
| Oecologia | Acceptance | 4190 | 207 | 230.487 |
| Oncoscience | Acceptance | 257 | 30 | 41.9883 |
| Open biology | Acceptance | 60 | 34 | 36.7833 |
| Pest management science | Acceptance | 1206 | 129 | 151.104 |
| Physiology and molecular biology of plants : an international journal of functional plant biology | Acceptance | 128 | 89 | 94.1719 |
| Plant and soil | Acceptance | 11 | 151 | 146.636 |
| Plant biology (Stuttgart, Germany) | Acceptance | 443 | 90 | 102.971 |
| Plant ecology | Acceptance | 1 | 71 | 71 |
| Plant molecular biology | Acceptance | 2207 | 117 | 136.86 |
| Plant molecular biology reporter / ISPMB | Acceptance | 1 | 94 | 94 |
| Plant science : an international journal of experimental plant biology | Acceptance | 1079 | 87 | 97.6793 |
| Plant systematics and evolution = Entwicklungsgeschichte und Systematik der Pflanzen | Acceptance | 1 | 51 | 51 |
| Plant, cell & environment | Acceptance | 598 | 98.5 | 133.808 |
| PLoS biology | Acceptance | 2064 | 141 | 150.992 |
| PLoS computational biology | Acceptance | 3820 | 148 | 162.955 |
| PloS one | Acceptance | 141494 | 108 | 125.857 |
| Polar biology | Acceptance | 2 | 151.5 | 151.5 |
| Powder technology | Acceptance | 4 | 200.5 | 208.5 |
| Primates; journal of primatology | Acceptance | 594 | 166.5 | 193.185 |
| Progress in biophysics and molecular biology | Acceptance | 382 | 84 | 103.893 |
| Radiation and environmental biophysics | Acceptance | 639 | 145 | 157.892 |
| Regional environmental change | Acceptance | 4 | 144.5 | 160 |
| Remote sensing of environment | Acceptance | 1 | 268 | 268 |
| Reproduction, fertility, and development | Acceptance | 1212 | 108 | 132.748 |
| Reproductive biology | Acceptance | 138 | 194.5 | 215.514 |
| Reproductive biology and endocrinology : RB&E | Acceptance | 1383 | 91 | 98.1208 |
| Reproductive medicine and biology | Acceptance | 14 | 58.5 | 72.2857 |
| Research in veterinary science | Acceptance | 2153 | 193 | 228.752 |
| Reviews in fish biology and fisheries | Acceptance | 1 | 91 | 91 |
| Reviews of environmental contamination and toxicology | Acceptance | 4 | 6 | 7.5 |
| Reviews on environmental health | Acceptance | 86 | 41 | 47.686 |
| Royal Society open science | Acceptance | 271 | 84 | 95.1107 |
| Science (New York, N.Y.) | Acceptance | 240 | 109 | 125.354 |
| Scientific reports | Acceptance | 18036 | 105 | 115.943 |
| Seminars in cell & developmental biology | Acceptance | 980 | 54 | 65.4153 |
| Sexual development : genetics, molecular biology, evolution, endocrinology, embryology, and pathology of sex determination and differentiation | Acceptance | 107 | 42 | 52.9252 |
| Soil biology & biochemistry | Acceptance | 20 | 122 | 139.45 |
| Systematic biology | Acceptance | 70 | 196.5 | 222.943 |
| The Annals of applied biology | Acceptance | 6 | 116 | 106.333 |
| The Canadian journal of infectious diseases & medical microbiology = Journal canadien des maladies infectieuses et de la microbiologie medicale / AMMI Canada | Acceptance | 102 | 118 | 137.431 |
| The international journal of biochemistry & cell biology | Acceptance | 2955 | 92 | 100.116 |
| The international journal of occupational and environmental medicine | Acceptance | 106 | 70 | 85.5189 |
| The Journal of agricultural science | Acceptance | 17 | 138 | 164.176 |
| The Journal of animal ecology | Acceptance | 493 | 181 | 197.081 |
| The Journal of applied ecology | Acceptance | 44 | 146.5 | 165.568 |
| The Journal of ecology | Acceptance | 18 | 124.5 | 144.556 |
| The Journal of experimental biology | Acceptance | 253 | 116 | 122.36 |
| The Journal of molluscan studies | Acceptance | 3 | 242 | 231 |
| The Journal of wildlife management | Acceptance | 8 | 325.5 | 325.375 |
| The open microbiology journal | Acceptance | 146 | 36 | 57.0274 |
| The Plant journal : for cell and molecular biology | Acceptance | 1050 | 91 | 120.608 |
| The Science of the total environment | Acceptance | 9985 | 106 | 123.749 |
| The Scientific World Journal | Acceptance | 6910 | 55 | 64.1708 |
| Theoretical biology & medical modelling | Acceptance | 483 | 89 | 105.52 |
| Theoretical ecology | Acceptance | 4 | 84 | 105.5 |
| Theoretical population biology | Acceptance | 664 | 172 | 210.696 |
| Theriogenology | Acceptance | 6386 | 146 | 173.388 |
| Toxicological and environmental chemistry | Acceptance | 1 | 306 | 306 |
| Trends in cell biology | Acceptance | 879 | 67 | 79.198 |
| Trends in ecology & evolution | Acceptance | 944 | 119 | 135.846 |
| Tropical plant biology | Acceptance | 8 | 67.5 | 72.625 |
| Ultrasound in medicine & biology | Acceptance | 2778 | 160 | 177.61 |
| Vaccine | Acceptance | 10793 | 107 | 127.23 |
| Weed research | Acceptance | 1 | 387 | 387 |
| Wilderness & environmental medicine | Acceptance | 361 | 160 | 161.931 |
| Wildlife Society bulletin | Acceptance | 1 | 198 | 198 |
| Wiley interdisciplinary reviews. Developmental biology | Acceptance | 70 | 99.5 | 151.071 |
| Wiley interdisciplinary reviews. Systems biology and medicine | Acceptance | 67 | 83 | 94.3881 |
| World journal of microbiology & biotechnology | Acceptance | 1573 | 93 | 110.743 |
| Zoo biology | Acceptance | 336 | 167 | 188.92 |
| Zoological journal of the Linnean Society | Acceptance | 4 | 154.5 | 180.5 |
| Zoological letters | Acceptance | 35 | 84 | 106.6 |
| Zoological science | Acceptance | 321 | 93 | 114.065 |
| Zoology (Jena, Germany) | Acceptance | 534 | 112 | 126.639 |
| Accident; analysis and prevention | Publication | 2683 | 27 | 31.6422 |
| Acta zoologica (Stockholm, Sweden) | Publication | 2 | 35 | 35 |
| Advances in enzyme regulation | Publication | 28 | 42.5 | 38.8571 |
| African journal of ecology | Publication | 1 | 41 | 41 |
| Agricultural and forest entomology | Publication | 1 | 115 | 115 |
| Ambio | Publication | 299 | 30 | 34.3378 |
| American journal of botany | Publication | 139 | 35 | 39.9209 |
| American journal of primatology | Publication | 548 | 38 | 43.7099 |
| Animal conservation | Publication | 1 | 61 | 61 |
| Animal: an international journal of animal bioscience | Publication | 1 | 78 | 78 |
| Annals of botany | Publication | 117 | 50 | 52.4444 |
| Annals of clinical microbiology and antimicrobials | Publication | 410 | 0 | 7.02683 |
| Annals of occupational and environmental medicine | Publication | 120 | 31.5 | 41.675 |
| Apidologie | Publication | 1 | 31 | 31 |
| Applied and environmental microbiology | Publication | 651 | 6 | 6.96621 |
| Applied entomology and zoology | Publication | 1 | 40 | 40 |
| Applied microbiology and biotechnology | Publication | 8049 | 27 | 36.279 |
| Aquatic conservation : marine and freshwater ecosystems | Publication | 1 | 78 | 78 |
| Aquatic ecology | Publication | 1 | 19 | 19 |
| Archives of environmental contamination and toxicology | Publication | 1584 | 30 | 66.524 |
| Archives of oral biology | Publication | 1854 | 31 | 35.1138 |
| Austral ecology | Publication | 1 | 86 | 86 |
| Behavioral ecology : official journal of the International Society for Behavioral Ecology | Publication | 57 | 44 | 51.1579 |
| Behavioral ecology and sociobiology | Publication | 98 | 22 | 26.4184 |
| Biochemistry and molecular biology education : a bimonthly publication of the International Union of Biochemistry and Molecular Biology | Publication | 173 | 52 | 60.3295 |
| Biodiversity and conservation | Publication | 6 | 14 | 18.6667 |
| BioEssays : news and reviews in molecular, cellular and developmental biology | Publication | 30 | 34.5 | 37.4 |
| Bioinformatics and biology insights | Publication | 46 | 55 | 69.1522 |
| Biological invasions | Publication | 5 | 10 | 23.8 |
| Biology & philosophy | Publication | 24 | 21.5 | 31.5 |
| Biology and fertility of soils | Publication | 2 | 18.5 | 18.5 |
| Biology direct | Publication | 481 | 0 | 5.98337 |
| Biology letters | Publication | 2 | 25 | 25 |
| Biology of reproduction | Publication | 187 | 8 | 9.3369 |
| Biology of the neonate | Publication | 233 | 87 | 101.082 |
| Biology open | Publication | 204 | 31.5 | 34.0833 |
| Biomedical engineering and computational biology | Publication | 2 | 32 | 32 |
| Bioresource technology | Publication | 12622 | 11 | 24.3634 |
| BMC biology | Publication | 838 | 0 | 5.72076 |
| BMC chemical biology | Publication | 39 | 0 | 3.12821 |
| BMC developmental biology | Publication | 832 | 0 | 3.32091 |
| BMC ecology | Publication | 307 | 0 | 8.19218 |
| BMC evolutionary biology | Publication | 2944 | 0 | 5.71671 |
| BMC molecular biology | Publication | 713 | 0 | 2.2216 |
| BMC plant biology | Publication | 2036 | 3 | 8.95285 |
| BMC structural biology | Publication | 481 | 0 | 2.56965 |
| BMC systems biology | Publication | 1173 | 0 | 8.28559 |
| Brazilian journal of biology = Revista brasleira de biologia | Publication | 178 | 488.5 | 483.23 |
| Brazilian journal of microbiology : [publication of the Brazilian Society for Microbiology] | Publication | 1187 | 209 | 217.799 |
| Bulletin of environmental contamination and toxicology | Publication | 2498 | 15 | 24.223 |
| Bulletin of mathematical biology | Publication | 1061 | 37 | 75.1084 |
| Cell biology and toxicology | Publication | 354 | 27 | 42.4831 |
| Cell biology international | Publication | 1197 | 28 | 38.6332 |
| Cellular & molecular biology letters | Publication | 359 | 14 | 39.1838 |
| Chemistry & biology | Publication | 296 | 41 | 43.1284 |
| Clinical microbiology and infection : the official publication of the European Society of Clinical Microbiology and Infectious Diseases | Publication | 735 | 38 | 40.302 |
| Communicative & integrative biology | Publication | 329 | 74 | 73.8389 |
| Comparative biochemistry and physiology. Part A, Molecular & integrative physiology | Publication | 2048 | 8 | 14.6831 |
| Comparative biochemistry and physiology. Part B, Biochemistry & molecular biology | Publication | 1373 | 8 | 14.5696 |
| Comparative immunology, microbiology and infectious diseases | Publication | 476 | 34.5 | 45.0462 |
| Computational biology and chemistry | Publication | 582 | 11 | 19.567 |
| Conservation biology : the journal of the Society for Conservation Biology | Publication | 512 | 120.5 | 127.992 |
| Conservation genetics (Print) | Publication | 1 | 22 | 22 |
| Conservation genetics resources | Publication | 3 | 13 | 75 |
| Cryobiology | Publication | 947 | 10 | 17.037 |
| Current biology : CB | Publication | 2752 | 36 | 37.1537 |
| Current issues in molecular biology | Publication | 10 | 105 | 107.3 |
| Current opinion in chemical biology | Publication | 921 | 28 | 29.6352 |
| Current opinion in plant biology | Publication | 989 | 26 | 28.0506 |
| Current opinion in structural biology | Publication | 942 | 26 | 29.5276 |
| Developmental biology | Publication | 4368 | 9 | 14.274 |
| Diagnostic microbiology and infectious disease | Publication | 1997 | 45 | 49.9194 |
| EcoHealth | Publication | 443 | 43 | 55.386 |
| Ecological entomology | Publication | 4 | 86.5 | 81.75 |
| Ecology and evolution | Publication | 1346 | 39 | 47.6642 |
| Ecology letters | Publication | 446 | 35 | 38.704 |
| Economics and human biology | Publication | 405 | 12 | 19.1037 |
| Ecosystems (New York, N.Y.) | Publication | 2 | 41.5 | 41.5 |
| Ecotoxicology and environmental safety | Publication | 2960 | 29 | 37.848 |
| Energy & environmental science | Publication | 2 | 23.5 | 23.5 |
| Entomologia experimentalis et applicata | Publication | 5 | 74 | 91.2 |
| Environment international | Publication | 1590 | 33 | 35.9082 |
| Environmental and molecular mutagenesis | Publication | 343 | 43 | 45.0408 |
| Environmental biology of fishes | Publication | 1 | 27 | 27 |
| Environmental biosafety research | Publication | 22 | 54.5 | 84.2273 |
| Environmental chemistry letters | Publication | 6 | 12.5 | 15.8333 |
| Environmental entomology | Publication | 173 | 35 | 43.948 |
| Environmental geochemistry and health | Publication | 701 | 28 | 67.475 |
| Environmental health : a global access science source | Publication | 847 | 0 | 6.87721 |
| Environmental health and preventive medicine | Publication | 356 | 25 | 28.3511 |
| Environmental health and toxicology | Publication | 87 | 50 | 59.1724 |
| Environmental health insights | Publication | 34 | 62 | 72.3529 |
| Environmental health perspectives | Publication | 1677 | 0 | 2.30173 |
| Environmental management | Publication | 1659 | 28 | 44.252 |
| Environmental microbiology | Publication | 837 | 41 | 48.264 |
| Environmental microbiology reports | Publication | 257 | 40 | 55.4786 |
| Environmental monitoring and assessment | Publication | 5783 | 25 | 47.6313 |
| Environmental pollution (Barking, Essex : 1987) | Publication | 4543 | 36 | 43.6784 |
| Environmental research | Publication | 1756 | 34 | 39.3502 |
| Environmental science & technology letters | Publication | 4 | 0 | 0 |
| Environmental science and pollution research international | Publication | 5707 | 17 | 22.6991 |
| Environmental toxicology | Publication | 499 | 41 | 55.6693 |
| Environmental toxicology and chemistry / SETAC | Publication | 1654 | 72 | 77.3912 |
| Environmental toxicology and pharmacology | Publication | 1571 | 9 | 14.7479 |
| Environmetrics | Publication | 5 | 42 | 43.6 |
| Ethology, ecology & evolution | Publication | 1 | 56 | 56 |
| European journal of clinical microbiology & infectious diseases : official publication of the European Society of Clinical Microbiology | Publication | 2055 | 24 | 29.4365 |
| European journal of microbiology & immunology | Publication | 146 | 70 | 73.1027 |
| European journal of soil science | Publication | 2 | 103 | 103 |
| Evolutionary biology | Publication | 20 | 16.5 | 19.65 |
| Evolutionary ecology | Publication | 1 | 17 | 17 |
| Experimental biology and medicine (Maywood, N.J.) | Publication | 130 | 58 | 63.4846 |
| FASEB journal : official publication of the Federation of American Societies for Experimental Biology | Publication | 432 | 16 | 19.9722 |
| FEMS microbiology ecology | Publication | 919 | 32 | 30.6725 |
| FEMS microbiology letters | Publication | 913 | 24 | 22.9715 |
| Folia primatologica; international journal of primatology | Publication | 166 | 88 | 94.8313 |
| Food and environmental virology | Publication | 151 | 13 | 14.404 |
| Freshwater biology | Publication | 3 | 40 | 40.3333 |
| Frontiers in zoology | Publication | 399 | 4 | 9.48622 |
| Functional ecology | Publication | 1 | 70 | 70 |
| Fungal biology | Publication | 623 | 12 | 14.9358 |
| Fungal genetics and biology : FG & B | Publication | 1186 | 11 | 18.4992 |
| Genesis (New York, N.Y. : 2000) | Publication | 358 | 38.5 | 52.1201 |
| Genetics and molecular biology | Publication | 610 | 139 | 143.338 |
| Genome biology and evolution | Publication | 54 | 6 | 9.48148 |
| Global change biology | Publication | 982 | 71 | 80.8116 |
| Global change biology. Bioenergy | Publication | 1 | 56 | 56 |
| Historical biology | Publication | 2 | 303.5 | 303.5 |
| Hormones and behavior | Publication | 1654 | 10 | 18.0599 |
| Hydrobiologia | Publication | 2 | 41.5 | 41.5 |
| In vitro cellular & developmental biology. Animal | Publication | 824 | 36 | 43.7245 |
| Infection ecology & epidemiology | Publication | 63 | 32 | 36.3968 |
| Insect biochemistry and molecular biology | Publication | 1108 | 12 | 14.6453 |
| Insect molecular biology | Publication | 10 | 53 | 53.1 |
| Insect science | Publication | 202 | 109 | 127.658 |
| Insectes sociaux | Publication | 14 | 23 | 24.8571 |
| Integrated environmental assessment and management | Publication | 270 | 93.5 | 100.4 |
| International archives of occupational and environmental health | Publication | 1327 | 24 | 48.5803 |
| International journal for parasitology. Parasites and wildlife | Publication | 126 | 15 | 16.7698 |
| International journal of biochemistry and molecular biology | Publication | 88 | 8 | 18.6136 |
| International journal of environmental analytical chemistry | Publication | 1 | 127 | 127 |
| International journal of environmental research and public health | Publication | 822 | 7 | 11.9453 |
| International journal of evolutionary biology | Publication | 151 | 62 | 62.1722 |
| International journal of hygiene and environmental health | Publication | 728 | 32 | 39.5728 |
| International journal of medical microbiology : IJMM | Publication | 550 | 46 | 46.2091 |
| International journal of occupational medicine and environmental health | Publication | 286 | 102 | 117.318 |
| International journal of primatology | Publication | 47 | 87 | 103.596 |
| International microbiology : the official journal of the Spanish Society for Microbiology | Publication | 47 | 77 | 86.383 |
| Iranian journal of environmental health science & engineering | Publication | 69 | 11 | 16.6522 |
| Journal of agricultural & environmental ethics | Publication | 1 | 24 | 24 |
| Journal of agricultural, biological, and environmental statistics | Publication | 2 | 34 | 34 |
| Journal of biology | Publication | 37 | 40 | 42.2432 |
| Journal of chemical biology | Publication | 80 | 20 | 23.025 |
| Journal of chemical ecology | Publication | 1523 | 24 | 39.8831 |
| Journal of comparative physiology. B, Biochemical, systemic, and environmental physiology | Publication | 1153 | 26 | 34.7216 |
| Journal of economic entomology | Publication | 325 | 31 | 44.6062 |
| Journal of environmental and public health | Publication | 297 | 49 | 52.404 |
| Journal of environmental health science & engineering | Publication | 277 | 16 | 26.3069 |
| Journal of environmental management | Publication | 4016 | 36 | 52.8474 |
| Journal of environmental radioactivity | Publication | 1890 | 35 | 47.7974 |
| Journal of environmental sciences (China) | Publication | 438 | 138 | 142.395 |
| Journal of ethnobiology and ethnomedicine | Publication | 541 | 2 | 12.1017 |
| Journal of evolutionary biology | Publication | 763 | 43 | 48.7536 |
| Journal of experimental zoology. Part A, Ecological genetics and physiology | Publication | 293 | 43 | 48.9625 |
| Journal of experimental zoology. Part B, Molecular and developmental evolution | Publication | 225 | 43 | 52.5289 |
| Journal of exposure science & environmental epidemiology | Publication | 204 | 69 | 72.3971 |
| Journal of fish biology | Publication | 517 | 63 | 66.6963 |
| Journal of fish diseases | Publication | 372 | 62 | 110.218 |
| Journal of industrial microbiology & biotechnology | Publication | 1891 | 24 | 33.8329 |
| Journal of insect science (Online) | Publication | 146 | 48.5 | 79.3836 |
| Journal of integrative plant biology | Publication | 193 | 71 | 77.6995 |
| Journal of invertebrate pathology | Publication | 1277 | 9 | 19.5685 |
| Journal of mammary gland biology and neoplasia | Publication | 237 | 18 | 19.7975 |
| Journal of mathematical biology | Publication | 3 | 22 | 22 |
| Journal of microbiology (Seoul, Korea) | Publication | 1005 | 111 | 105.448 |
| Journal of molecular biology | Publication | 7025 | 8 | 11.3977 |
| Journal of molecular cell biology | Publication | 49 | 70 | 72.1224 |
| Journal of molecular microbiology and biotechnology | Publication | 9 | 73 | 79.1111 |
| Journal of pest science | Publication | 17 | 16 | 17.4706 |
| Journal of pharmacological and toxicological methods | Publication | 670 | 11 | 19.6522 |
| Journal of photochemistry and photobiology. B, Biology | Publication | 1679 | 10 | 17.4556 |
| Journal of soils and sediments | Publication | 1 | 25 | 25 |
| Journal of structural biology | Publication | 1785 | 9 | 13.6532 |
| Journal of theoretical biology | Publication | 4251 | 10 | 16.2997 |
| Journal of thermal biology | Publication | 222 | 5 | 7.1982 |
| Journal of trace elements in medicine and biology : organ of the Society for Minerals and Trace Elements (GMS) | Publication | 600 | 36 | 40.8217 |
| Journal of zoology (London, England : 1987) | Publication | 2 | 46 | 46 |
| Kinetoplastid biology and disease | Publication | 46 | 0 | 0 |
| Landscape ecology | Publication | 4 | 17 | 18.75 |
| Marine biology | Publication | 77 | 20 | 21.6494 |
| Marine ecology (Berlin, Germany) | Publication | 1 | 161 | 161 |
| Marine environmental research | Publication | 980 | 9 | 13.5204 |
| Marine pollution bulletin | Publication | 3682 | 28 | 33.1942 |
| Mathematical medicine and biology : a journal of the IMA | Publication | 17 | 43 | 42.9412 |
| Matrix biology : journal of the International Society for Matrix Biology | Publication | 641 | 11 | 17.1778 |
| Medical and veterinary entomology | Publication | 120 | 103 | 115.017 |
| Medical microbiology and immunology | Publication | 220 | 17 | 25.8 |
| Medical mycology case reports | Publication | 149 | 9 | 10.7852 |
| Methods in ecology and evolution / British Ecological Society | Publication | 13 | 50 | 53 |
| Microbial ecology | Publication | 2106 | 33 | 94.1804 |
| Microbial ecology in health and disease | Publication | 21 | 32 | 41.6667 |
| Microbiology (Reading, England) | Publication | 43 | 6 | 14.907 |
| Microbiology insights | Publication | 13 | 40 | 43.6154 |
| Molecular and cellular biology | Publication | 227 | 8 | 10.4714 |
| Molecular biology and evolution | Publication | 10 | 10 | 9.3 |
| Molecular biology international | Publication | 95 | 57 | 57.2842 |
| Molecular biology of the cell | Publication | 268 | 7 | 7.40672 |
| Molecular biology reports | Publication | 4673 | 13 | 20.0591 |
| Molecular ecology | Publication | 1171 | 43 | 47.3177 |
| Molecular ecology resources | Publication | 413 | 34 | 36.3269 |
| Molecular systems biology | Publication | 270 | 49.5 | 52.3074 |
| Movement ecology | Publication | 65 | 33 | 40.3077 |
| Mutation research. Genetic toxicology and environmental mutagenesis | Publication | 262 | 8 | 19.0076 |
| Mycorrhiza | Publication | 823 | 28 | 39.0158 |
| Nature | Publication | 4588 | 44 | 43.7544 |
| Nature chemical biology | Publication | 932 | 39 | 44.4796 |
| Nature structural & molecular biology | Publication | 1878 | 39 | 42.9835 |
| Nature structural biology | Publication | 53 | 38 | 40.3208 |
| Neurobiology of disease | Publication | 2458 | 10 | 18.2522 |
| Neurobiology of learning and memory | Publication | 1300 | 12 | 22.5438 |
| Nitric oxide : biology and chemistry / official journal of the Nitric Oxide Society | Publication | 799 | 9 | 15.4631 |
| Occupational and environmental medicine | Publication | 117 | 23 | 31.4188 |
| Oecologia | Publication | 4149 | 29 | 38.5527 |
| Oncoscience | Publication | 262 | 3 | 4.76336 |
| Pest management science | Publication | 1058 | 52 | 59.7968 |
| Physiological and biochemical zoology : PBZ | Publication | 256 | 171.5 | 180.918 |
| Physiological entomology | Publication | 3 | 52 | 60.6667 |
| Physiology and molecular biology of plants : an international journal of functional plant biology | Publication | 128 | 18 | 25.4297 |
| Plant and soil | Publication | 11 | 17 | 22.2727 |
| Plant biology (Stuttgart, Germany) | Publication | 398 | 66.5 | 71.1407 |
| Plant ecology | Publication | 1 | 60 | 60 |
| Plant molecular biology | Publication | 1376 | 17 | 25.0167 |
| Plant molecular biology reporter / ISPMB | Publication | 1 | 35 | 35 |
| Plant science : an international journal of experimental plant biology | Publication | 1077 | 8 | 10.2136 |
| Plant systematics and evolution = Entwicklungsgeschichte und Systematik der Pflanzen | Publication | 1 | 36 | 36 |
| Plant, cell & environment | Publication | 545 | 49 | 55.3725 |
| PLoS biology | Publication | 1502 | 43 | 48.771 |
| PLoS computational biology | Publication | 3531 | 49 | 52.3948 |
| PloS one | Publication | 138699 | 35 | 38.8 |
| Polar biology | Publication | 2 | 21.5 | 21.5 |
| Primates; journal of primatology | Publication | 576 | 34 | 53.8594 |
| Progress in biophysics and molecular biology | Publication | 390 | 10 | 12.7026 |
| Radiation and environmental biophysics | Publication | 640 | 22 | 30.3016 |
| Regional environmental change | Publication | 4 | 51.5 | 69 |
| Reproductive biology | Publication | 138 | 15 | 58.1522 |
| Reproductive biology and endocrinology : RB&E | Publication | 1383 | 0 | 4.63268 |
| Reproductive medicine and biology | Publication | 14 | 26 | 25.3571 |
| Research in veterinary science | Publication | 2113 | 30 | 35.23 |
| Reviews in fish biology and fisheries | Publication | 1 | 41 | 41 |
| Royal Society open science | Publication | 271 | 29 | 30.1439 |
| Science (New York, N.Y.) | Publication | 240 | 16 | 23.3208 |
| Scientific reports | Publication | 1232 | 22 | 27.2589 |
| Seminars in cell & developmental biology | Publication | 962 | 8 | 9.91892 |
| Sexual development : genetics, molecular biology, evolution, endocrinology, embryology, and pathology of sex determination and differentiation | Publication | 206 | 98 | 114.913 |
| Systematic biology | Publication | 71 | 8 | 17.2535 |
| The Annals of applied biology | Publication | 6 | 62 | 62.5 |
| The international journal of biochemistry & cell biology | Publication | 2626 | 9 | 12.917 |
| The Journal of agricultural science | Publication | 16 | 45 | 65.1875 |
| The Journal of animal ecology | Publication | 454 | 52 | 56.0529 |
| The Journal of applied ecology | Publication | 31 | 42 | 42.4516 |
| The Journal of ecology | Publication | 13 | 62 | 65.4615 |
| The Journal of experimental biology | Publication | 253 | 14 | 14.6403 |
| The Journal of molluscan studies | Publication | 3 | 75 | 75.6667 |
| The Journal of wildlife management | Publication | 8 | 79.5 | 79.5 |
| The open microbiology journal | Publication | 145 | 53 | 61.3172 |
| The Plant journal : for cell and molecular biology | Publication | 837 | 45 | 47.4922 |
| The Science of the total environment | Publication | 9150 | 30 | 35.6903 |
| The Scientific World Journal | Publication | 6984 | 46 | 60.4218 |
| Theoretical biology & medical modelling | Publication | 487 | 0 | 6.29363 |
| Theoretical ecology | Publication | 4 | 33.5 | 33.75 |
| Theoretical population biology | Publication | 631 | 13 | 20.8811 |
| Theriogenology | Publication | 3256 | 49 | 49.5875 |
| Toxicological and environmental chemistry | Publication | 2 | 37.5 | 37.5 |
| Trends in cell biology | Publication | 804 | 31 | 32.7948 |
| Trends in ecology & evolution | Publication | 869 | 29 | 36.8228 |
| Tropical plant biology | Publication | 8 | 32.5 | 31.375 |
| Ultrasound in medicine & biology | Publication | 1892 | 72 | 75.5788 |
| Vaccine | Publication | 9459 | 15 | 18.7637 |
| Weed research | Publication | 1 | 76 | 76 |
| Wilderness & environmental medicine | Publication | 272 | 88 | 84.2904 |
| Wildlife Society bulletin | Publication | 1 | 142 | 142 |
| Wiley interdisciplinary reviews. Developmental biology | Publication | 66 | 43 | 45.9242 |
| Wiley interdisciplinary reviews. Systems biology and medicine | Publication | 64 | 40.5 | 40.5 |
| World journal of microbiology & biotechnology | Publication | 1228 | 11 | 15.5407 |
| Zoo biology | Publication | 326 | 39.5 | 47.4693 |
| Zoologica scripta | Publication | 1 | 44 | 44 |
| Zoological journal of the Linnean Society | Publication | 3 | 126 | 116 |
| Zoological letters | Publication | 35 | 44 | 69.1143 |
| Zoology (Jena, Germany) | Publication | 427 | 128 | 128.564 |
