# Supplementary material for: Reducing publication delay to improve the efficiency and impact of conservation science
Source: PeerJ. 2021 Oct 12;9:e12245. doi: 10.7717/peerj.12245 (PMC8519180; doi:10.7717/peerj.12245)
Supplement: Supplemental Information 5 — EMMs values and 95% CIs are presented in Fig. 1 (Main Text) and were derived from a quasi–Poisson GLM (see Methods in Main Text) with three explanatory variables (synopsis, publication date, and peer-review category). The number of studies for each synopsis may not match the Conservation Evidence website as the database is being dynamically updated with more studies over time. In addition, studies can be present in multiple synopses, and for the purposes of our analyses we also excluded reviews and meta-analyses, as well as studies with no end date of data collection. [file peerj-09-12245-s005.docx]

Table S2 — Mean publication delay (Estimated Marginal Means (EMMs)), associated 95% Confidence Intervals (CI), and numbers of studies for each Conservation Evidence synopsis (a collection of studies based on the conservation subject in which interventions have been tested). EMMs values and 95% CIs are presented in Figure 1 (Main Text) and were derived from a quasi-Poisson GLM (see Methods in Main Text) with three explanatory variables (synopsis, publication date, and peer-review category). The number of studies for each synopsis may not match the Conservation Evidence website as the database is being dynamically updated with more studies over time. In addition, studies can be present in multiple synopses, and for the purposes of our analyses we also excluded reviews and meta-analyses, as well as studies with no end date of data collection.

| **Synopsis** | **Mean** | **SE** | **Lower 95% CI** | **Upper 95% CI** | **Number of studies** |
| --- | --- | --- | --- | --- | --- |
| Bee Conservation | 1.41 | 0.14 | 1.04 | 1.90 | 569 |
| Sustainable Aquaculture | 1.41 | 0.22 | 0.88 | 2.25 | 237 |
| Management of Captive Animals | 1.60 | 0.20 | 1.11 | 2.31 | 182 |
| Amphibian Conservation | 1.74 | 0.10 | 1.48 | 2.06 | 1584 |
| Control of Freshwater Invasive Species | 1.85 | 0.20 | 1.35 | 2.54 | 121 |
| Bat Conservation | 2.20 | 0.16 | 1.77 | 2.74 | 996 |
| Primate Conservation | 2.41 | 0.15 | 2.01 | 2.88 | 405 |
| Farmland Conservation | 2.43 | 0.10 | 2.14 | 2.75 | 101 |
| Shrubland and Heathland Conservation | 2.75 | 0.19 | 2.23 | 3.38 | 738 |
| Bird Conservation | 2.80 | 0.11 | 2.49 | 3.14 | 172 |
| Mediterranean Farmland | 2.86 | 0.13 | 2.49 | 3.28 | 284 |
| Natural Pest Control | 2.87 | 0.22 | 2.29 | 3.59 | 343 |
| Peatland Conservation | 2.89 | 0.17 | 2.42 | 3.46 | 214 |
| Terrestrial Mammal Conservation | 2.93 | 0.12 | 2.60 | 3.30 | 197 |
| Soil Fertility | 3.11 | 0.21 | 2.54 | 3.82 | 231 |
| Subtidal Benthic Invertebrate Conservation | 3.20 | 0.20 | 2.65 | 3.87 | 67 |
| Forest Conservation | 3.33 | 0.17 | 2.85 | 3.89 | 1208 |
