# Supplementary material for: Reducing publication delay to improve the efficiency and impact of conservation science
Source: PeerJ. 2021 Oct 12;9:e12245. doi: 10.7717/peerj.12245 (PMC8519180; doi:10.7717/peerj.12245)
Supplement: Supplemental Information 6 — Selected model was the full one with all three explanatory variables of peer-review, publication date, and synopsis. Publication delay (difference between year data collection ended and year study was published) was the response variable. [file peerj-09-12245-s006.docx]

Table S3 — Selection of model structure by qAIC ranking. Selected model was the full one with all three explanatory variables of peer-review, publication date, and synopsis. Publication delay (difference between year data collection ended and year study was published) was the response variable.

| Model rank | Model structure | df | logLik | qAIC | delta |
| --- | --- | --- | --- | --- | --- |
| Selected | Delay ~ peer-review + publication date + synopsis | 19 | -18593 | 13070 | 0 |
| A | Delay ~ peer-review + synopsis | 18 | -18623 | 13089 | 18.5 |
| B | Delay ~ publication date + synopsis | 18 | -18668 | 13121 | 50.3 |
| C | Delay ~ synopsis | 17 | -18700 | 13141 | 70.8 |
| D | Delay ~ peer-review + publication date | 3 | -19001 | 13324 | 253.3 |
| E | Delay ~ peer-review | 2 | -19044 | 13352 | 281.9 |
| F | Delay ~ publication date | 2 | -19130 | 13413 | 342.3 |
| G | Delay ~ 1 | 1 | -19181 | 13446 | 375.7 |
