# Supplementary material for: Reducing publication delay to improve the efficiency and impact of conservation science
Source: PeerJ. 2021 Oct 12;9:e12245. doi: 10.7717/peerj.12245 (PMC8519180; doi:10.7717/peerj.12245)
Supplement: Supplemental Information 8 — Selected model for all taxa combined (amphibians, birds, and mammals) was the full one with all four explanatory variables of peer-review, publication date, synopsis, and IUCN Red List category. Selected models for amphibians and mammals included peer-review and publication date, whilst the selected model for birds only included IUCN Red List category. Publication delay (difference between year data collection ended and year study was published) was the response variable for all models. Models within <2 units of qAIC were decided between by selecting the most simple model structure (i.e., selecting the most parsimonious model). [file peerj-09-12245-s008.docx]

Table S5 — Selection of model structure by qAIC ranking for taxonomic analyses. Selected model for all taxa combined (amphibians, birds, and mammals) was the full one with all four explanatory variables of peer-review, publication date, synopsis, and IUCN Red List category. Selected models for amphibians and mammals included peer-review and publication date, whilst the selected model for birds only included IUCN Red List category. Publication delay (difference between year data collection ended and year study was published) was the response variable for all models. Models within <2 units of qAIC were decided between by selecting the most simple model structure (i.e., selecting the most parsimonious model).

| **All taxa (amphibians, birds, and mammals)** | | | | | |
| --- | --- | --- | --- | --- | --- |
| Model rank | Model structure | df | logLik | qAIC | delta |
| Selected | Delay ~ peer-review + publication date + synopsis + IUCN Red List category | 11 | -20068 | 11038 | 0 |
| A | Delay ~ peer-review + synopsis + IUCN Red List category | 10 | -20105 | 11057 | 18.3 |
| B | Delay ~ publication date + synopsis + IUCN Red List category | 10 | -20153 | 11083 | 44.8 |
| C | Delay ~ synopsis + IUCN Red List category | 9 | -20195 | 11105 | 66.1 |
| D | Delay ~ peer-review + publication date + synopsis | 7 | -20345 | 11183 | 144.5 |
| E | Delay ~ peer-review + synopsis | 6 | -20386 | 11203 | 164.6 |
| F | Delay ~ publication date + synopsis | 6 | -20437 | 11231 | 192.9 |
| G | Delay ~ synopsis | 5 | -20484 | 11255 | 216.5 |
| H | Delay ~ peer-review + publication date + IUCN Red List category | 7 | -20497 | 11266 | 227.8 |
| I | Delay ~ peer-review + IUCN Red List category | 6 | -20531 | 11283 | 244.3 |
| J | Delay ~ publication date + IUCN Red List category | 6 | -20630 | 11337 | 298.9 |
| K | Delay ~ IUCN Red List category | 5 | -20668 | 11356 | 317.5 |
| L | Delay ~ peer-review + publication date | 3 | -20798 | 11423 | 384.6 |
| M | Delay ~ peer-review | 2 | -20841 | 11445 | 406.5 |
| N | Delay ~ publication date | 2 | -20929 | 11493 | 454.8 |
| O | Delay ~ 1 | 1 | -20977 | 11517 | 478.9 |
| **Amphibians** | | | | | |
| Model rank | Model structure | df | logLik | qAIC | delta |
| Selected | Delay ~ peer-review + publication date | 3 | -1586 | 1340 | 0 |
| A | Delay ~ publication date | 2 | -1595 | 1345 | 5.1 |
| B | Delay ~ peer-review | 2 | -1595 | 1346 | 5.8 |
| C | Delay ~ peer-review + publication date + IUCN Red List category | 7 | -1585 | 1347 | 6.8 |
| D | Delay ~ 1 | 1 | -1604 | 1351 | 11.1 |
| E | Delay ~ peer-review + IUCN Red List category | 6 | -1593 | 1351 | 11.4 |
| F | Delay ~ publication date + IUCN Red List category | 6 | -1593 | 1352 | 12.1 |
| G | Delay ~ IUCN Red List category | 5 | -1602 | 1357 | 17.2 |
| **Birds** | | | | | |
| Model rank | Model structure | df | logLik | qAIC | delta |
| Selected | Delay ~ IUCN Red List category | 5 | -8424 | 5162 | 0 |
| A | Delay ~ publication date + IUCN Red List category | 6 | -8423 | 5163 | 1.3 |
| B | Delay ~ peer-review + IUCN Red List category | 6 | -8423 | 5164 | 1.6 |
| C | Delay ~ peer-review + publication date + IUCN Red List category | 7 | -8422 | 5165 | 2.9 |
| D | Delay ~ 1 | 1 | -8521 | 5214 | 51.4 |
| E | Delay ~ publication date | 2 | -8521 | 5215 | 53.3 |
| F | Delay ~ peer-review | 2 | -8521 | 5216 | 53.3 |
| G | Delay ~ peer-review + publication date | 3 | -8521 | 5217 | 55.3 |
| **Mammals** | | | | | |
| Model rank | Model structure | df | logLik | qAIC | delta |
| Selected | Delay ~ peer-review + publication date + synopsis + IUCN Red List category | 9 | -9946 | 4700 | 0 |
| A | Delay ~ peer-review + synopsis + IUCN Red List category | 8 | -9990 | 4719 | 18.7 |
| B | Delay ~ publication date + synopsis + IUCN Red List category | 8 | -10043 | 4744 | 43.5 |
| C | Delay ~ synopsis + IUCN Red List category | 7 | -10099 | 4768 | 68 |
| D | Delay ~ peer-review + publication date + synopsis | 5 | -10166 | 4796 | 95.4 |
| E | Delay ~ peer-review + publication date + IUCN Red List category | 7 | -10196 | 4814 | 113.3 |
| F | Delay ~ peer-review + synopsis | 4 | -10223 | 4820 | 120 |
| G | Delay ~ peer-review + IUCN Red List category | 6 | -10236 | 4831 | 130.4 |
| H | Delay ~ publication date + synopsis | 4 | -10287 | 4851 | 150.2 |
| I | Delay ~ publication date + IUCN Red List category | 6 | -10291 | 4857 | 156.4 |
| J | Delay ~ IUCN Red List category | 5 | -10347 | 4881 | 180.3 |
| K | Delay ~ synopsis | 3 | -10359 | 4882 | 182.1 |
| L | Delay ~ peer-review + publication date | 3 | -10390 | 4897 | 196.9 |
| M | Delay ~ peer-review | 2 | -10422 | 4910 | 210 |
| N | Delay ~ publication date | 2 | -10501 | 4947 | 246.8 |
| O | Delay ~ 1 | 1 | -10545 | 4966 | 265.9 |
