# Supplementary material for: Reducing publication delay to improve the efficiency and impact of conservation science
Source: PeerJ. 2021 Oct 12;9:e12245. doi: 10.7717/peerj.12245 (PMC8519180; doi:10.7717/peerj.12245)
Supplement: Supplemental Information 9 — The synopsis ‘Management of Captive Animals’ and publication source ‘non-peer-reviewed’ were set to the zero value of the beta slope and used as reference categories as these had the lowest mean publication delay values based on preliminary exploration of the data. p-values of 0.000 represent p < 0.001. [file peerj-09-12245-s009.docx]

Table S6 — Results of quasi-Poisson Generalised Linear Model (GLM) (see Methods) for taxonomic sensitivity analyses (selecting a random species from each study rather than the most threatened species). The synopsis ‘Management of Captive Animals’ and publication source ‘non-peer-reviewed’ were set to the zero value of the beta slope and used as reference categories as these had the lowest mean publication delay values based on preliminary exploration of the data. p-values of 0.000 represent p<0.001.

| Amphibians, birds, and mammals | | | | |
| --- | --- | --- | --- | --- |
| Parameter | Estimate | Standard Error | t-value | p-value |
| Intercept | -11.604 | 2.755 | -4.212 | 0 |
| Publication Date | 0.006 | 0.001 | 4.445 | 0 |
| Terrestrial Mammal Conservation | 0.132 | 0.029 | 4.582 | 0 |
| Bat Conservation | -0.281 | 0.049 | -5.716 | 0 |
| Amphibian Conservation | -0.492 | 0.053 | -9.214 | 0 |
| Primate Conservation | -0.315 | 0.064 | -4.942 | 0 |
| Peer-reviewed (yes) | 0.532 | 0.085 | 6.293 | 0 |
| Near Threatened | -0.025 | 0.039 | -0.641 | 0.522 |
| Vulnerable | -0.058 | 0.04 | -1.448 | 0.148 |
| Endangered | 0.427 | 0.036 | 11.926 | 0 |
| Critically Endangered | 0.189 | 0.061 | 3.126 | 0.002 |
| Amphibians only | | | | |
| Parameter | Estimate | Standard Error | t-value | p-value |
| Intercept | -31.018 | 12.398 | -2.502 | 0.013 |
| Publication Date | 0.016 | 0.006 | 2.533 | 0.012 |
| Peer-reviewed (yes) | 0.334 | 0.128 | 2.607 | 0.009 |
| Near Threatened | 0.221 | 0.209 | 1.057 | 0.291 |
| Vulnerable | 0.024 | 0.136 | 0.18 | 0.857 |
| Endangered | 0.024 | 0.149 | 0.164 | 0.87 |
| Critically Endangered | 0.079 | 0.156 | 0.507 | 0.612 |
| Birds only | | | | |
| Parameter | Estimate | Standard Error | t-value | p-value |
| Intercept | -1.976 | 3.715 | -0.532 | 0.595 |
| Near Threatened | 0.002 | 0.002 | 0.833 | 0.405 |
| Vulnerable | 0.085 | 0.139 | 0.616 | 0.538 |
| Endangered | -0.031 | 0.046 | -0.678 | 0.498 |
| Critically Endangered | -0.249 | 0.071 | -3.514 | 0.000 |
| Mammals only | | | | |
| Parameter | Estimate | Standard Error | t-value | p-value |
| Intercept | -18.86 | 4.319 | -4.367 | 0.000 |
| Publication Date | 0.010 | 0.002 | 4.461 | 0.000 |
| Peer-reviewed (yes) | 0.885 | 0.152 | 5.829 | 0.000 |
| Near Threatened | -0.021 | 0.073 | -0.284 | 0.776 |
| Vulnerable | 0.027 | 0.054 | 0.490 | 0.624 |
| Endangered | 0.453 | 0.046 | 9.847 | 0.000 |
| Critically Endangered | 0.330 | 0.081 | 4.081 | 0.000 |
| Bat Conservation | -0.431 | 0.052 | -8.260 | 0.000 |
| Primate Conservation | -0.488 | 0.071 | -6.872 | 0.000 |
