# Supplementary material for: Reducing publication delay to improve the efficiency and impact of conservation science
Source: PeerJ. 2021 Oct 12;9:e12245. doi: 10.7717/peerj.12245 (PMC8519180; doi:10.7717/peerj.12245)
Supplement: Supplemental Information 11 — The synopsis ‘Bee Conservation’ and publication source ‘non-peer-reviewed’ were set to the zero value of the beta slope and used as reference categories (see Methods). p-values of 0.000 represent p < 0.001. [file peerj-09-12245-s011.docx]

Table S8 — Results of quasi-Poisson Generalised Linear Model (GLM) to test for predictors of publication delay. The synopsis ‘Bee Conservation’ and publication source ‘non-peer-reviewed’ were set to the zero value of the beta slope and used as reference categories (see Methods). p-values of 0.000 represent p<0.001.

| Parameter | Estimate | Standard Error | t-value | p-value |
| --- | --- | --- | --- | --- |
| Intercept | -11.361 | 2.569 | -4.422 | 0.000 |
| Publication date | 0.006 | 0.001 | 4.464 | 0.000 |
| Bird Conservation | 0.686 | 0.099 | 6.938 | 0.000 |
| Farmland Conservation | 0.544 | 0.101 | 5.374 | 0.000 |
| Natural Pest Control | 0.711 | 0.119 | 5.985 | 0.000 |
| Control of Freshwater Invasive Species | 0.271 | 0.142 | 1.911 | 0.056 |
| Shrubland and Heathland Conservation | 0.668 | 0.115 | 5.826 | 0.000 |
| Terrestrial Mammal Conservation | 0.732 | 0.100 | 7.355 | 0.000 |
| Bat Conservation | 0.447 | 0.117 | 3.829 | 0.000 |
| Amphibian Conservation | 0.213 | 0.108 | 1.968 | 0.049 |
| Forest Conservation | 0.861 | 0.105 | 8.216 | 0.000 |
| Primate Conservation | 0.535 | 0.110 | 4.872 | 0.000 |
| Peatland Conservation | 0.719 | 0.110 | 6.518 | 0.000 |
| Mediterranean Farmland | 0.707 | 0.102 | 6.925 | 0.000 |
| Subtidal Benthic Invertebrate Conservation | 0.821 | 0.111 | 7.380 | 0.000 |
| Management of Captive Animals | 0.130 | 0.153 | 0.852 | 0.394 |
| Soil Fertility | 0.794 | 0.114 | 6.966 | 0.000 |
| Sustainable Aquaculture | 0.003 | 0.182 | 0.014 | 0.989 |
| Peer-review (Yes) | 0.441 | 0.065 | 6.779 | 0.000 |
